# The Budding Yeast Cdc48^Shp1^ Complex Promotes Cell Cycle Progression by Positive Regulation of Protein Phosphatase 1 (Glc7)

**DOI:** 10.1371/journal.pone.0056486

**Published:** 2013-02-13

**Authors:** Stefanie Böhm, Alexander Buchberger

**Affiliations:** Department of Biochemistry, Biocenter, University of Würzburg, Würzburg, Germany; Florida State University, United States of America

## Abstract

The conserved, ubiquitin-selective AAA ATPase Cdc48 regulates numerous cellular processes including protein quality control, DNA repair and the cell cycle. Cdc48 function is tightly controlled by a multitude of cofactors mediating substrate specificity and processing. The UBX domain protein Shp1 is a *bona fide* substrate-recruiting cofactor of Cdc48 in the budding yeast *S. cerevisiae*. Even though Shp1 has been proposed to be a positive regulator of Glc7, the catalytic subunit of protein phosphatase 1 in *S. cerevisiae*, its cellular functions in complex with Cdc48 remain largely unknown. Here we show that deletion of the *SHP1* gene results in severe growth defects and a cell cycle delay at the metaphase to anaphase transition caused by reduced Glc7 activity. Using an engineered Cdc48 binding-deficient variant of Shp1, we establish the Cdc48^Shp1^ complex as a critical regulator of mitotic Glc7 activity. We demonstrate that *shp1* mutants possess a perturbed balance of Glc7 phosphatase and Ipl1 (Aurora B) kinase activities and show that hyper-phosphorylation of the kinetochore protein Dam1, a key mitotic substrate of Glc7 and Ipl1, is a critical defect in *shp1*. We also show for the first time a physical interaction between Glc7 and Shp1 *in vivo*. Whereas loss of Shp1 does not significantly affect Glc7 protein levels or localization, it causes reduced binding of the activator protein Glc8 to Glc7. Our data suggest that the Cdc48^Shp1^ complex controls Glc7 activity by regulating its interaction with Glc8 and possibly further regulatory subunits.

## Introduction

The evolutionarily conserved and highly abundant eukaryotic AAA (ATPase associated with various cellular activities) ATPase Cdc48 (also known as TER94 in *Drosophila* and as p97 and VCP in mammals) has emerged as an important motor and regulator for the turnover of ubiquitylated proteins [1]–[4]. It converts chemical energy released by ATP hydrolysis into mechanical force in order to drive the segregation of ubiquitylated substrate proteins from stable protein complexes, membranes, and chromatin [1], [3]–[5]. Cdc48 plays central roles in the proteasomal degradation of protein quality control targets, cell cycle regulators, and transcription factors [3], [4], [6]. Recently, Cdc48 has also been implicated in the lysosomal degradation of proteins delivered *via* autophagic and endosomal pathways [3], [4], [7]. Thus, Cdc48 is involved in the three major routes of regulated intracellular proteolysis in eukaryotes. In addition, Cdc48 has been shown to function in non-proteolytic processes in the fusion of homotypic membrane vesicles of the Endoplasmic Reticulum, Golgi apparatus, and the nuclear envelope [8], [9].

The involvement of Cdc48 in such diverse cellular processes requires tight control of its activity. Indeed, a large number of cofactor proteins regulate central aspects of Cdc48 function, including its subcellular localization and substrate specificity [2], [3], [10]. The mutual exclusive binding of two major cofactors, the heterodimer Ufd1-Npl4 and Shp1 (also known as p47 in vertebrates), defines two distinct Cdc48 complexes, Cdc48^Ufd1-Npl4^ and Cdc48^Shp1^, which are specialized in proteasomal and non-proteasomal pathways, respectively [10]–[12]. Cofactor binding to Cdc48 appears to be hierarchical, as additional cofactors bind to the Cdc48^Ufd1-Npl4^ and Cdc48^Shp1^ complexes in order to further fine-tune their cellular function [10], [13]. Cofactors interact with Cdc48 by virtue of one or more Cdc48 binding modules, among them the ubiquitin-like UBX domain [10], [14]–[16] and the linear binding site 1 (BS1) motif (also known as SHP box) [17]–[19]. UBX domain containing proteins constitute the largest family of Cdc48 cofactors [10]. In the budding yeast *Saccharomyces cerevisiae*, seven UBX proteins were identified and shown to bind Cdc48 [20], [21]: Shp1 itself (also known as Ubx1) and Ubx2 through Ubx7. In addition to their carboxyl-terminal UBX domain, Shp1, Ubx2 and Ubx5 possess an amino-terminal UBA domain mediating the binding of ubiquitin and ubiquitylated substrates [20], [22]–[24], and thus exhibit the prototypical architecture of substrate-recruiting adaptors for Cdc48 [20], [25], [26]. So far, cellular functions were identified for only few of the yeast UBX proteins and include roles in ER-associated protein degradation [22], [27], [28], lipid droplet homeostasis [29], and UV-induced turnover of RNA polymerase II [24]. By contrast, the role of Shp1 is still poorly understood. Shp1 has been implicated in the proteasomal degradation of a Cdc48 model substrate [20], but the physiological relevance of this finding remains unclear. More recently, Shp1 has been shown to bind the autophagy factor Atg8 and to be involved in autophagosome biogenesis [30]. However, the severe phenotypes of *shp1* mutants suggest that Shp1 has additional, more critical cellular functions [20], [31].

The *SHP1* gene was first identified in a genetic screen for suppressors of the otherwise lethal over-expression of *GLC7*, the sole catalytic subunit of protein phosphatase 1 (PP1) in yeast [32]. Two *shp1* (suppressor of high-copy PP1) alleles tolerated the overexpression of *GLC7* and, in turn, exhibited phenotypes reminiscent of *glc7* loss-of-function mutants. *shp1* null mutants are inviable in the W303 strain background [31] and have reduced PP1 activity in other backgrounds [32], [33], consistent with the model that Shp1 is a positive regulator required for normal Glc7 activity [32]–[34]. However, the mechanism by which Shp1 influences Glc7 activity is unknown. It has been proposed that Shp1 positively affects Glc7 activity by a yet undefined indirect mechanism [32]–[34] or by controlling the nuclear localization of Glc7 [31].

Glc7 regulates numerous cellular processes including glycogen metabolism, glucose repression, RNA processing, meiosis and sporulation, DNA damage recovery, actin organization, cell wall morphogenesis, and mitosis (reviewed in [34], [35]). A mitotic function of PP1 was first discovered in the fission yeast *S. pombe* [36], [37] and subsequently also shown to exist in higher eukaryotes such as *Drosophila* and mammals [38], [39]. In *S. cerevisiae*, PP1 is crucial for proper chromosome segregation and, consequently, several different *glc7* mutants have been shown to arrest at or before anaphase onset [40]–[42].

Accurate distribution of the replicated genome during cell division is essential for viability and depends on proper chromosome segregation. During mitosis, two physically connected sister chromatids must be faithfully segregated to mother and daughter cell, an event controlled by the spindle assembly checkpoint (SAC) [43], [44]. In order for the yeast metaphase to anaphase transition to occur, each kinetochore must attach to a single microtubule of the mitotic spindle [43]–[45]. The SAC prevents anaphase onset by keeping the APC/C^Cdc20^ ubiquitin ligase complex inactive. Once proper bi-polar attachment is achieved, active APC/C^Cdc20^ ubiquitylates the mitotic substrate Pds1 (securin), which in turn is rapidly degraded by the 26S proteasome resulting in cleavage of cohesin and sister-chromatid separation [43], [44], [46]–[48].

The mitotic delay of *glc7-129* and *glc7-10* mutants depends on the SAC [49], [50]. During mitosis, Glc7 has been described to oppose the kinase activity of Ipl1 (Aurora B) [51] by dephosphorylating the kinetochore proteins Ndc10 and Dam1, as well as histone H3 [50], [52]–[56]. The correct balance of the Glc7 phosphatase and Ipl1 kinase activities ensures proper chromosome bi-orientation. According to the prevalent model, Ipl1 senses incorrect attachments lacking tension during metaphase and phosphorylates a critical kinetochore component, Dam1. Glc7 then reverses this modification and thereby allows microtubule (re-)attachment. This eventually leads to correct bi-polar attachment and cell cycle progression [34], [57], [58]. Consequently, certain *glc7* partial-loss-of-function alleles suppress the temperature sensitivity of hypomorphic *ipl1* mutants by restoring the phosphatase to kinase balance [53], [58], [59].

Shp1 has previously been implicated in the regulation of several cytosolic functions of Glc7 [32], [33], [60]–[62]. In this study, we identify the Cdc48^Shp1^ complex as a critical positive regulator of Glc7 activity towards mitotic Ipl1 substrates including Dam1. We show that *shp1* mutants exhibit a SAC-mediated cell cycle delay resulting from reduced Glc7 activity, which in turn is caused by the lack of a specific Cdc48^Shp1^ function. Moreover, we provide evidence that Cdc48^Shp1^ regulates Glc7 activity by controlling its interaction with regulatory subunits rather than affecting Glc7 protein levels or localization.

## Results

### *shp1* mutants are impaired in growth and mitotic progression

**Figure 1:**
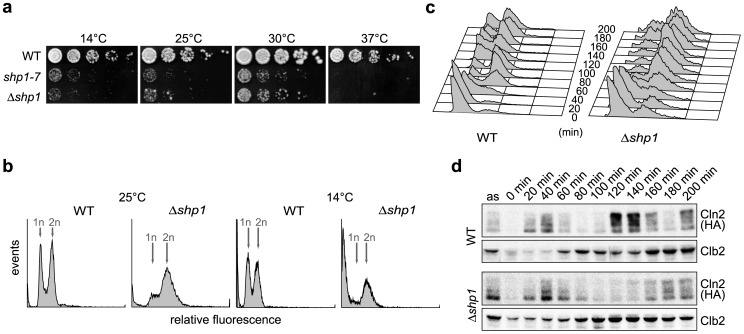
*shp1* null mutants exhibit growth defects and mitotic delay. (a) *shp1* null mutants are cold- and temperature-sensitive. 5-fold serial dilutions of wild-type (WT), *shp1-7* and *Δshp1* cultures were spotted on YPD plates and incubated at the indicated temperatures for 3 days. (b) *shp1* null cells accumulate and terminally arrest in G2/M at 25°C and 14°C, respectively. Asynchronously growing WT and *Δshp1* cultures at 25°C were split and incubated for 14 h at 25°C or 14°C as indicated. Cells were fixed and analyzed for DNA content by staining with propidium iodide and flow cytometry. The peaks for single (1n) and double (2n) DNA content are labeled. (c, d) *shp1* null cells are delayed in mitotic progression. Exponentially growing WT and *Δshp1* strains expressing *CLN2^3HA^* were arrested in G1 with α-factor and released. Samples were taken every 20 min. (c) FACS analysis was performed as in (b). (d) Clb2 and Cln2^3HA^ levels were analyzed by Western blot.

In order to study cellular functions of Shp1, we generated *shp1* null mutants completely lacking Shp1 in the DF5 strain background by mutating the start codon (*shp1-7*) or by deleting the entire coding region (*Δshp1*). In contrast to the W303 background, where *SHP1* is essential (data not shown; [31]), DF5 *shp1* null mutants are viable but grow slowly and are hypersensitive against multiple stress conditions, including high and low temperature and various cell-damaging agents (Fig. 1a) [20]. In addition to these growth phenotypes, *shp1* null cells also exhibit an altered cell cycle distribution (Fig. 1b). Analysis of the DNA content of asynchronous cultures by flow cytometry showed a significant accumulation of cells with 2n DNA content in *Δshp1* compared to the wild-type. At 25°C, the proportion of wild-type cells in the G1/S and G2/M cell cycle phases was nearly equal (43±3% and 56±3%, respectively), whereas *Δshp1* cultures contained only 27±6% cells in G1/S, but 73±6% in G2/M. At the non-permissive temperature of 14°C, *Δshp1* cultures contained a majority of cells in G2/M, hardly any cells in G1/S, and a significant amount of sub-G1 material potentially indicating the presence of inviable cell remnants (Fig. 1b).

The cell cycle delay was further analyzed by releasing wild-type and *Δshp1* cultures at 25°C from a G1 cell cycle arrest induced by the mating pheromone α-factor (Fig. 1cd). The FACS profiles of samples taken at various time points after release show that wild-type and *Δshp1* cells both entered G2/M approximately 60–80 min after release (Fig. 1c). However, whereas wild-type cells initiated G1 of the following cell cycle after about 120 min, the number of *Δshp1* cells in G1 started to increase only after 160 to 180 min. This G2/M delay of *Δshp1* was confirmed by the analysis of cyclin levels by Western blot (Fig. 1d). As judged by the degradation of the G1/S cyclin Cln2 and the onset of expression of the mitotic cyclin Clb2, wild-type and *Δshp1* strains both entered G2/M 60–80 min after release. Wild-type cells initiated the next cell cycle at about 120 min, as indicated by the decrease in Clb2 and increase in Cln2 levels. By contrast, the majority of *Δshp1* cells remained in G2/M with high Clb2 levels and undetectable Cln2 levels until 160 min after release. Note that the increased Clb2 levels observed in α-factor arrested *Δshp1* cells are not caused by defective mitotic exit resulting in G1 entry with high Clb2 levels (data not shown), but are due to a less efficient G1 arrest observed in *shp1* mutants (see Fig. 1c). In summary, our data show that Shp1 is required for normal mitotic progression.

### Shp1 functions in growth and mitotic progression require Cdc48 binding

All known cellular functions of Shp1 and its mammalian homologue p47 are believed to be based on its role as an adaptor of Cdc48/p97 [8], [10], [20], [30], [31], suggesting that the mitotic phenotype of *shp1* null mutants described above may involve Cdc48 as well. However, Cdc48 is essential, and conditional *cdc48* mutants exhibit pleiotropic phenotypes including defects at several stages of the cell cycle [63]–[67], thus complicating a meaningful interpretation with respect to Shp1-dependent mitotic defects.

**Figure 2:**
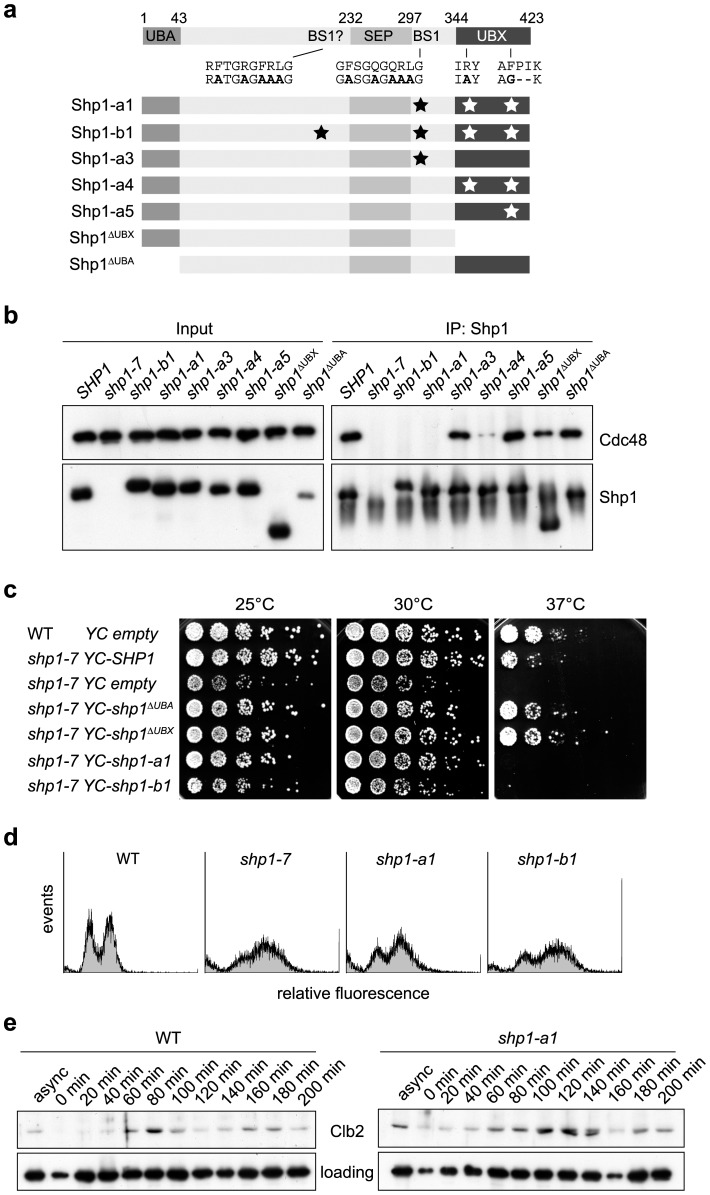
Shp1 functions in growth and mitotic progression require Cdc48 binding. (a) Schematic of *shp1* mutations in Cdc48 binding motifs engineered for this study. Wild-type Shp1 is shown at the top, with defined domains and motifs labeled. UBA, ubiquitin-associated domain; SEP, Shp1, eyc, and p47 domain; UBX, ubiquitin regulatory X domain; BS1, binding site 1. Key BS1 and UBX residues mutated in Cdc48 binding-deficient *shp1* alleles are indicated in bold in the sequence and by asterisks in the outlines of the Shp1 variant proteins shown below. (b) Simultaneous mutation of the R-FPR motif in the UBX domain and of binding site(s) 1 abolishes Cdc48 binding *in vivo*. Lysates of cells expressing the indicated *shp1* alleles were subjected to immunoprecipitation with a Shp1 antibody and analyzed for Cdc48 co-immunoprecipitation by Western blot. (c) *shp1* mutants defective in Cdc48 binding are temperature sensitive. Wild-type (WT) and *shp1-7* mutant cells carrying the indicated centromeric plasmids were analyzed for growth at the indicated temperatures as described for Fig. 1a. (d, e) *shp1* mutants defective in Cdc48 binding are delayed in mitotic progression. Asynchronous WT and *shp1-a1* cultures were analyzed by FACS (d) as described in the legend to Fig. 1b, and WT and *shp1-a1* cultures synchronized by α-factor arrest/release were analyzed by Western blot against the mitotic cyclin Clb2 (e) as described in the legend to Fig. 1d.

To overcome the limitations of conditional *cdc48* alleles, we engineered *shp1* alleles encoding Shp1 variants specifically impaired in Cdc48 binding. To this end, sets of amino acid residues in the UBX domain and the binding site 1 (BS1) motif of Shp1 critical for Cdc48 binding were mutated separately and in combinations (Fig. 2a). In addition, key residues in a potential second BS1 motif preceding the SEP domain (Kay Hofmann and A.B., unpublished) were also mutated. Finally, deletion variants lacking the entire UBX and UBA domain, respectively, were constructed. All *shp1* alleles were introduced into DF5 by chromosomal integration in single copy under control of the *SHP1* promoter. As expected from previous reports [18], [68], deletion of the UBA domain did not interfere with Cdc48 binding at all, and deletion of the entire UBX domain or separate mutation of UBX domain or BS1 residues resulted only in partial loss of Cdc48 binding in immunoprecipitation experiments (Fig. 2b). In contrast, the simultaneous mutation of key residues in the UBX domain and in one or both BS1 motifs in the *shp1-a1* and *shp1-b1* alleles led to a complete loss of Cdc48 binding. Phenotypic analysis showed that both alleles confer temperature sensitivity, indicating that this *shp1* phenotype involves Cdc48 binding (Fig. 2c). Next, we analyzed the *shp1-a1* and *shp1-b1* mutants for potential mitotic defects. Intriguingly, like the *shp1* null mutants, the FACS profiles of the Cdc48 binding-deficient mutants revealed an accumulation of cells in G2/M (Fig. 2d), and a delayed mitotic progression was observed with elevated Clb2 levels until 180–200 min after release from G1 arrest (Fig. 2e). These results demonstrate for the first time that the mitotic defects of *shp1* mutants are due to the lack of a specific, Shp1-mediated function(s) of Cdc48 during cell cycle progression.

### The mitotic delay of *shp1* mutants involves SAC activation

**Figure 3:**
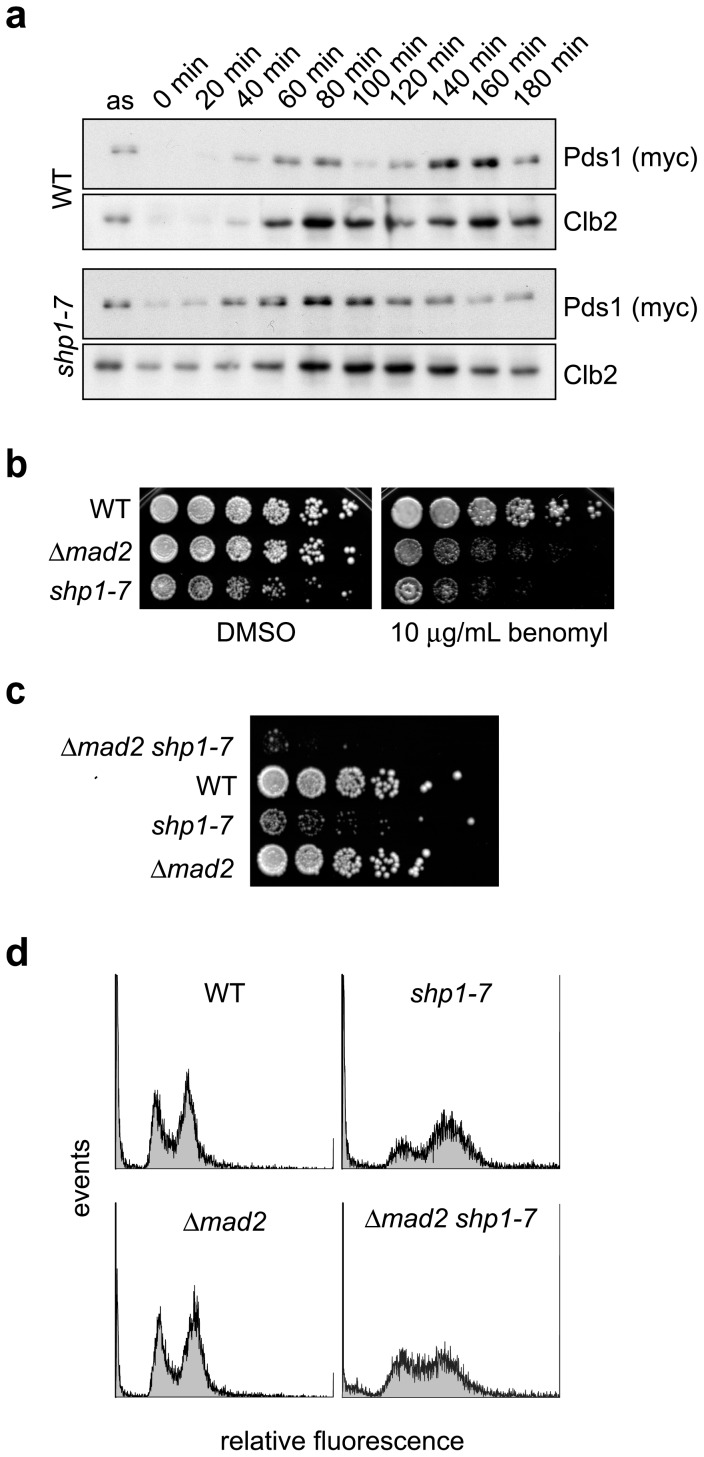
The cell cycle delay of *shp1* mutants is caused by SAC activation. (a) Delayed degradation of Pds1 (securin). Wild-type (WT) and *shp1-7* null mutant cultures were synchronized by α-factor arrest/release and analyzed by Western blot against Pds1^18myc^ as described in the legend to Fig. 1d. (b) *shp1-*7 is hypersensitive towards the spindle poison benomyl. Growth of WT, *shp1-7* and *Δmad2* cells at 25°C in the absence (DMSO) and presence of benomyl was analyzed as described for Fig. 1a. (c) Synthetic growth defect of *shp1-7 Δmad2*. Haploid progeny of one tetrad from the cross of *shp1-7* with *Δmad2* was analyzed for growth at 25°C as described for Fig. 1a. (d) The mitotic delay of *shp1-7* is alleviated by checkpoint inactivation. The cell cycle distribution of the indicated strains at 25°C was analyzed by FACS as described in the legend to Fig. 1b.

The metaphase to anaphase transition is controlled by the spindle assembly checkpoint (SAC) through inhibition of the APC/C^Cdc20^ ubiquitin ligase complex until chromosome bi-orientation is achieved [43], [44], [69]. In order to test if the early mitotic delay of *shp1* mutants is the result of SAC activation, we analyzed the stability of Pds1 (budding yeast securin) in wild-type and *shp1-7* cultures released from G1 arrest (Fig. 3a). Pds1 was expressed approximately 40 min after the release both in wild-type and *shp1-7*. However, whereas Pds1 was completely degraded 100 min after release in wild-type, it was significantly stabilized and detectable until the end of the time course in *shp1-7*. These results indicate a prolonged SAC activation in *shp1* and pinpoint the mitotic delay of *shp1* to the metaphase to anaphase transition.

Mutants with spindle or kinetochore defects are hypersensitive to microtubule depolymerizing agents [69]–[72] and often depend on the presence of an intact SAC for viability [73]–[75]. Consistent with the observed SAC activation, *shp1-7* was indeed found to be hypersensitive towards benomyl (Fig. 3b). Furthermore, we detected a strong negative genetic interaction approaching synthetic lethality between *shp1-7* and a deletion mutant of a central SAC component, *Δmad2* (Fig. 3c). Of note, surviving *shp1-7 Δmad2* cells displayed a more even G1/S *versus* G2/M distribution than the *shp1-7* single mutant (Fig. 3d), further supporting the notion that the mitotic delay of *shp1-7* is caused by SAC activation.

### The mitotic phenotype of *shp1* mutants is caused by reduced Glc7 activity

**Figure 4:**
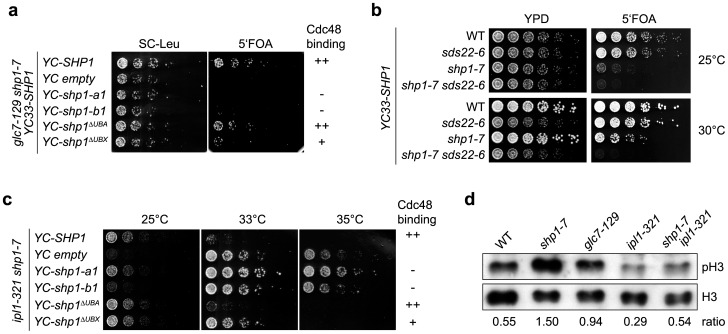
*shp1* mutants exhibit reduced Glc7 activity. (a) Synthetic lethality of *glc7-129* with *shp1* mutants defective in Cdc48 binding. *glc7-129 shp1-7* double mutant cells carrying the *URA3*-based *SHP1* expression plasmid *YC33-SHP1* and a *LEU2*-based centromeric plasmid for the expression of the indicated wild-type and mutant *SHP1* alleles were spotted in serial dilutions onto control plates (SC-Leu) or plates containing 5-fluoro orotic acid (5′FOA) to counterselect against *YC33-SHP1*. The ability of the *shp1* mutant gene products to bind Cdc48 as shown in Fig. 2b is indicated at the right. (b) Synthetic lethality of *shp1-7* and *sds22-6*. Growth of haploid progeny of one tetrad from the cross of *shp1-7* with *sds22-6* carrying *YC-SHP1* was analyzed on control (YPD) and 5′FOA plates as described above. (c) Positive genetic interaction between *ipl1-321* and *shp1* mutants defective in Cdc48 binding. Growth of haploid progeny of one tetrad from the cross of *shp1-7* with *ipl1-321* carrying a centromeric plasmid for the expression of the indicated wild-type and mutant *SHP1* alleles was analyzed at the indicated temperatures. The ability of the *shp1* mutant gene products to bind Cdc48 is indicated at the right. (d) Hyper-phosphorylation of histone H3 in *shp1-7*. The phosphorylation state of histone H3 in the indicated WT and mutant strains at 35°C was analyzed by Western blot using an antibody recognizing phosphorylated residue Ser10 (pH3) and total H3, respectively. The ratio of the signal intensities (pH3/total H3) is given at the bottom.

*shp1* mutants were originally identified based on their ability to tolerate elevated Glc7 levels [32], and Shp1 has been proposed to be a positive regulator of Glc7 [32]–[35]. To test if the mitotic phenotype of *shp1* mutants is related to Glc7 function(s), we analyzed genetic interactions between *shp1* and the conditional *glc7* allele, *glc7-129*, which at the non-permissive temperature confers a cell cycle arrest at the metaphase-anaphase transition [76]. Furthermore, the mitotic arrest of *glc7-129* was reported to depend on the SAC [49]. Intriguingly, the *shp1-7 glc7-129* double mutant was inviable at all temperatures tested (Fig. S1a and data not shown), indicating overlapping cellular functions of Shp1 and Glc7. As expected, the synthetic lethality of *shp1-7 glc7-129* could be suppressed by a centromeric plasmid encoding wild-type *SHP1* (Fig. 4a). Importantly, when we tested the Cdc48 binding-deficient alleles *shp1^ΔUBX^*, *shp1-a1*, and *shp1-b1*, their ability to suppress the lethality of *shp1-7 glc7-129* correlated with the ability of the respective gene products to bind Cdc48, demonstrating that an intact Cdc48^Shp1^ complex is required for the viability of *glc7-129*.

To confirm that Shp1 is involved in mitotic functions of Glc7, we next tested genetic interactions between *SHP1* and the major nuclear Glc7 regulatory subunit, *SDS22* (Fig. 4b). Indeed, we observed synthetic lethality of the *shp1-7 sds22-6* double mutant as well, strongly suggesting that Shp1 is critical for a mitotic function(s) of Glc7. Finally, we analyzed genetic interactions between *SHP1* and *IPL1*, the gene encoding the yeast Aurora B kinase homologue. Ipl1 has been described to antagonize mitotic functions of Glc7 at the kinetochore, and the correct balance of Ipl1 kinase and Glc7 phosphatase activities is crucial for unperturbed mitotic progression [50], [52], [55], [59], [77]. Importantly, we observed a clear mutual suppression of the *shp1-7* and *ipl1-321* temperature sensitivities. The *shp1-7 ipl1-321* double mutant grew better at 33°C and 37°C than either single mutant (Fig. S1b), suggesting that reduced Ipl1 activity partially alleviates the defects caused by reduced Glc7 activity in *shp1-7*. However, this positive genetic interaction between *shp1* and *ipl1-321* was not observed for Shp1 variants proficient in Cdc48 binding (Fig. 4c), again confirming that Shp1 regulates mitotic Glc7 activity in its capacity as a Cdc48 cofactor. Together, our genetic analysis strongly suggests that *shp1* mutants possess limiting mitotic Glc7 activity leading to unbalanced Ipl1 activity.

To directly address if nuclear substrates of Glc7 are hyper-phosphorylated in *shp1*, the phosphorylation state of histone H3 in wild-type and *shp1*, *glc7*, and *ipl1* mutants was analyzed at the non-permissive temperature of 35°C (Fig. 4d). Residue S10 of histone H3 is phosphorylated by Ipl1 during mitosis, and the phosphorylation level is governed by the balance of Ipl1 kinase and Glc7 phosphatase activities [53]. As expected, the *glc7-129* and *ipl1-321* mutants exhibited increased and decreased phosphorylation of histone H3, respectively. In *shp1-7* cells, the phosphorylated form was strikingly increased, directly demonstrating that Glc7 phosphatase activity is impaired in *shp1-7*. Importantly, the accumulation of phosphorylated histone H3 in *shp1-*7 was efficiently suppressed in the *ipl1-321 shp1-*7 double mutant, resulting in a normal ratio of phosphorylated and total histone H3. Thus, Shp1 indeed controls the balance of Ipl1 and Glc7 activities towards their nuclear target, histone H3.

**Figure 5:**
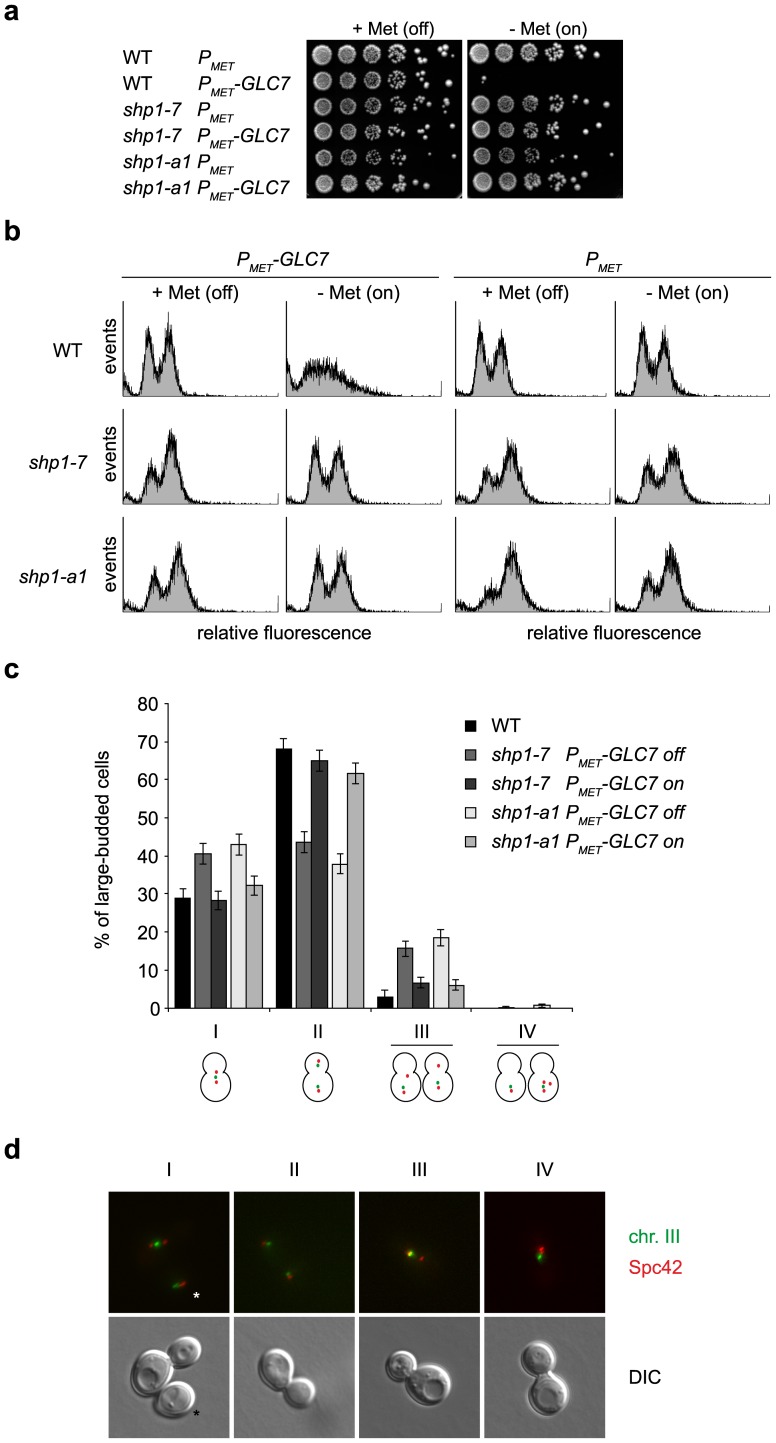
The mitotic phenotype of *shp1* mutants is caused by reduced Glc7 activity. (a) *shp1* mutants tolerate over-expression of *GLC7*. Wild-type (WT) and *shp1-7* and *shp1-a1* mutant cells expressing *GLC7* from an integrative plasmid under the control of the inducible *P_MET25_* promoter (*P_MET_-GLC7*) were analyzed for growth at 25°C in the presence (+Met (off)) and absence (−Met (on)) of methionine in the growth medium. The respective strains carrying an empty integrative plasmid (*P_MET_*) served as control. (b) Over-expression of *GLC7* suppresses the mitotic delay of *shp1* mutants. The strains described in panel (a) were analyzed for cell cycle distribution by FACS in the absence and presence of methionine as indicated. (c) Over-expression of *GLC7* suppresses the chromosome segregation defect of *shp1* mutants. Sister chromatid separation of wild-type, *shp1-7* and *shp1-a1* mutant cells expressing *GLC7* under the control of the inducible *P_MET25_* promoter was analyzed at 25°C in the presence (*P_MET_-GLC7* off) and absence (*P_MET_-GLC7* on) of methionine in the growth medium. Large-budded cells (n>300 for each condition) were sorted into four classes based on the relative orientation of the ^GFP^LacI-marked chromosomes III and the spindle pole body (SPB) marker Spc42^Mars^: I, normal metaphase spindle; II, normal anaphase spindle; III, meta-/anaphase spindle with segregation defect; IV, aberrant number of SPBs. Error bars indicate binomial standard errors. The distribution of the five cell types over the four classes is non-random with high statistic significance according to a Pearson's chi-squared test of independence (Χ^2^_(12)_ = 123.931; p>0.001). All pairwise differences within classes I–III between (i) wild-type and *shp1* mutants without over-expression of *GLC7*, and (ii) *shp1* mutants with and without *GLC7* over-expression are statistically significant with p<0.01 according to Fisher's exact test. (d) Representative examples of large-budded cells falling into the four classes analyzed in panel (c). Upper row, fluorescence microscopy of ^GFP^LacI-marked chromosomes III (chr. III) and Spc42^Mars^-marked SPBs; lower row, differential interference contrast (DIC) microscopy. The asterisks mark an additional unbudded cell in class I that was not included in the analysis.

We next tested if the supposed limiting Glc7 activity in *shp1* mutants can be overcome by raising cellular Glc7 levels. To this end, wild-type *GLC7* was expressed under the control of the strong, inducible *MET25* promoter (Fig. 5a). As expected [32], induction of *GLC7* over-expression on methionine-free medium was toxic for wild-type, but not *shp1-7*. Importantly, the Cdc48 binding-deficient allele *shp1-a1* also tolerated *GLC7* over-expression (Fig. 5a), again indicating that regulation of Glc7 activity by Shp1 is Cdc48-dependent. FACS analysis of wild-type cells confirmed that *GLC7* over-expression was highly toxic (Fig. 5b, top row). Remarkably, however, *GLC7* over-expression was not only tolerated by the *shp1-7* and *shp1-a1* mutants, but in fact suppressed the G2/M accumulation of the mutant cells (Fig. 5b, middle and bottom rows). Upon *GLC7* over-expression, the cell cycle distribution of *shp1-7* (46% G1/S, 53% G2/M) and *shp1-a1* cells (42% G1/S, 57% G2/M) approached that of wild-type cells without *GLC7* over-expression (43% G1/S, 54% G2/M).

Unbalanced Ipl1 and Glc7 activities give rise to chromosome segregation defects [50], [53], [59], suggesting that *shp1* mutants may be impaired in chromosome segregation as well. Indeed, yeast cells depleted of Shp1 were recently shown to exhibit defective chromosome bi-orientation [31]. Using strains containing a *lacO* array integrated at the *LEU2* locus of chromosome III and expressing ^GFP^LacI and the spindle pole body marker Spc42^Mars^, we analyzed chromosome segregation in wild-type and *shp1* mutants by live-cell fluorescence microscopy (Fig. 5cd). Compared to wild-type, cultures of *shp1-7* and *shp1-a1* contained significantly more large budded cells with a short spindle and unseparated chromosomes III, and significantly less cells with a long spindle and two separated chromosomes III (Fig. 5c). This finding is fully consistent with the metaphase to anaphase delay described above. Of note, *shp1-7* and *shp1-a1* also showed a significant increase in cells with chromosome segregation defects (15–20% of large-budded cells in comparison to 3% in the wild-type), as well as some aberrant spindles, confirming that Shp1 is required for faithful chromosome segregation. Importantly, and in line with the FACS data shown in Fig. 5b, over-expression of *GLC7* in the *shp1* mutants suppressed both the metaphase to anaphase delay and the chromosome segregation defects.

Taken together, these results demonstrate for the first time that nuclear Glc7 activity is reduced in *shp1* and that the mitotic phenotype of *shp1* results from limiting Glc7 activity.

### Dam1 hyper-phosphorylation causes growth defects of *shp1* mutants

**Figure 6:**
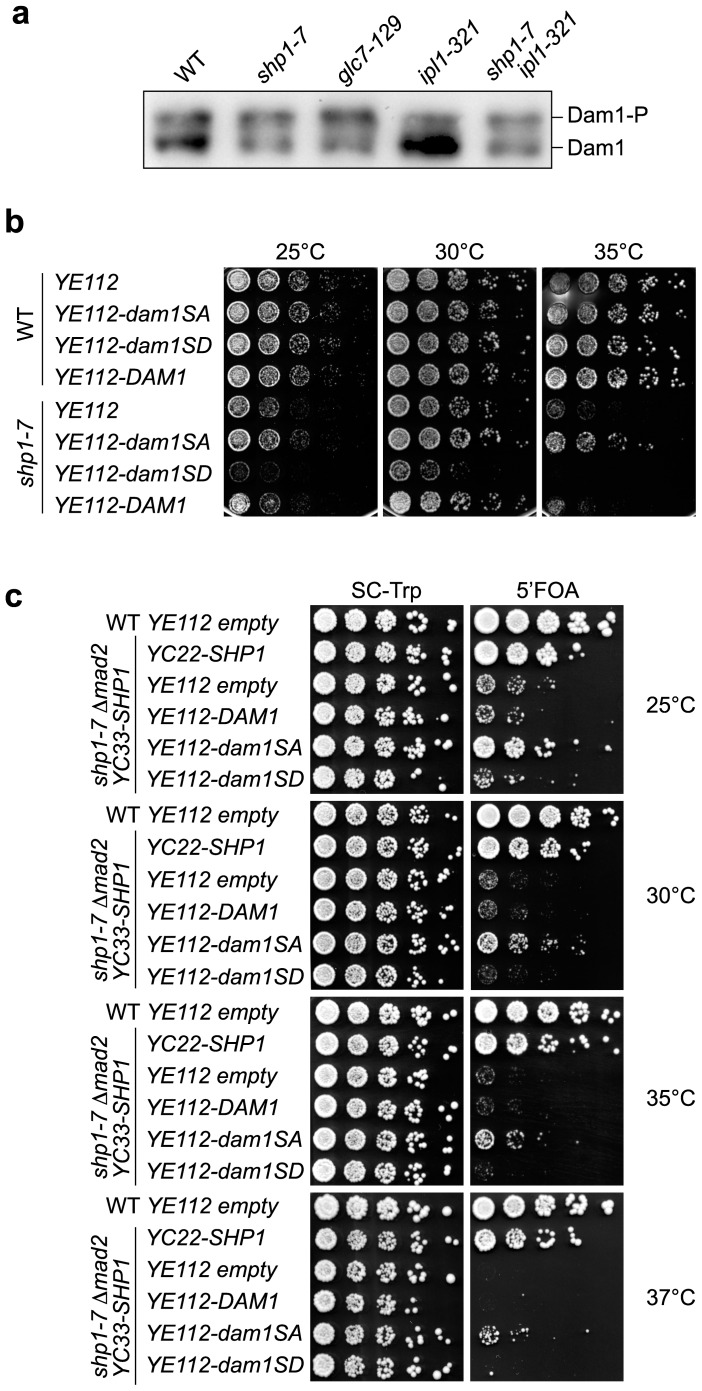
Dam1 hyper-phosphorylation is critical for the impaired growth of *shp1*. (a) Hyper-phosphorylation of Dam1 in *shp1-7*. The phosphorylation state of Dam1^9myc^ in the indicated wild-type (WT) and mutant strains at 35°C was analyzed by Western blot against the myc epitope tag. The position of Dam1 and phosphorylated Dam1 (Dam1-P) is indicated. (b) Phosphorylation-deficient and phosphorylation-mimicking *dam1* mutants suppress and exacerbate, respectively, the growth phenotype of *shp1-7*. Growth of WT and *shp1-7* cells carrying a *TRP1*-based high copy number plasmid (YE112) for the over-expression of the indicated wild-type and mutant *DAM1* alleles was analyzed on SC-Trp plates. (c) Expression of phosphorylation-deficient Dam1 suppresses the synthetic growth defect of *shp1-7 Δmad2*. *shp1-7 Δmad2* double mutant cells carrying *YC33-SHP1* and the indicated YE112 plasmids were spotted in serial dilutions onto control plates (SC-Trp) or plates containing 5-fluoro orotic acid (5′FOA) to counterselect against *YC33-SHP1*.

The phosphorylation state of the kinetochore protein Dam1 is critical for proper microtubule attachments during mitosis [55], [78]–[80]. Since Dam1 has been identified as a common substrate of Ipl1 kinase and Glc7 phosphatase activities [54]–[56], [81], [82], we next analyzed the phosphorylation state of Dam1 in *shp1* mutants. To this end, *shp1*, *glc7* and *ipl1* mutants were shifted to 35°C, and phosphorylation of Dam1 was analyzed by Western blot (Fig. 6a). Compared to wild-type cells, Dam1 was indeed hyper-phosphorylated in *shp1-7*, as judged by the reduction of the faster migrating non-phosphorylated form and the relative increase of the slower migrating phosphorylated form of Dam1. Of note, the increase of Dam1 phosphorylation in *shp1* was comparable to that observed in *glc7-129* cells. As expected, *ipl1-321* cells exhibited strongly reduced Dam1 phosphorylation under these conditions. It has previously been shown that the hypo-phosphorylation of Dam1 in *ipl1* mutants can be partially suppressed by a reduction of Glc7 phosphatase activity in *glc7* mutants [56]. In line with the reduced mitotic Glc7 activity in *shp1*, the *shp1-7 ipl1-321* double mutant indeed exhibited a roughly equal distribution of phosphorylated and non-phosphorylated Dam1 (Fig. 6a).

To elucidate if the hyper-phosphorylation of Dam1 in *shp1* mutants was responsible for the observed growth defects, we made use of previously described phosphorylation site mutants of Dam1 [55]. To this end, we transformed *shp1-7* with high copy number plasmids carrying wild-type *DAM1* or *dam1* mutated in residues S20 and S292, major target sites for Ipl1 [55], [82]. Whereas neither the empty vector control nor wild-type *DAM1* had an influence on the growth of *shp1-7*, over-expression of the *dam1SA* phospho-mutant incapable of being phosphorylated on residues 20 and 292 enabled *shp1-7* cells to grow robustly at 30 and 35°C (Fig. 6b) and, albeit very weakly, at 37°C (data not shown). Conversely, over-expression of the *dam1SD* mutant mimicking constitutive phosphorylation of residues 20 and 292 was detrimental for the growth of *shp1-7* at all temperatures (Fig. 6b). These data show for the first time that Dam1 is a critical target of unbalanced Ipl1 and Glc7 activities in *shp1* mutants.

We next tested if reduced Dam1 phosphorylation could also ameliorate the more severe growth defect of *shp1* in the absence of an intact SAC, *i.e.* in the *shp1-7 Δmad2* double mutant (Fig. 6c). Indeed, over-expression of the *dam1SA* phospho-mutant was able to partially suppress the growth defect of *shp1-7 Δmad2* both at ambient temperature and at the non-permissive temperature of 37°C. These results suggest that a reduction of phosphorylated Ipl1 target sites on Dam1 is sufficient to partially restore productive kinetochore-microtubule attachments and to limit chromosome mis-segregation in *shp1-7* to an extent that significantly improves viability.

### The nuclear localization of Glc7 is intact in *shp1* mutants

**Figure 7:**
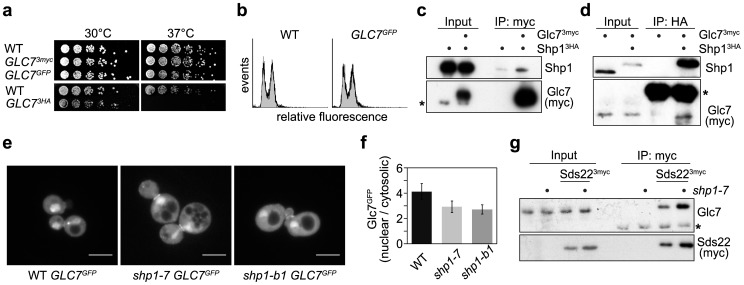
Glc7 nuclear localization is not severely affected in *shp1* mutants. (a, b) Functionality of epitope-tagged Glc7 variants. Growth of wild-type (WT) and strains expressing the indicated carboxyl-terminally epitope-tagged Glc7 fusion proteins from the chromosomal *GLC7* locus as the sole source of Glc7 was analyzed by (a) plate assay and (b) FACS. (c, d) Physical interaction between Glc7 and Shp1. (c) Lysates of strains expressing Shp1^3HA^ and Glc7^3myc^ as indicated were subjected to immunoprecipitation (IP) with anti-myc antibody and analyzed for co-precipitation of Shp1^3HA^. The asterisk marks a cross-reactive band of the Glc7 antibody. (d) Lysates of strains expressing Glc7^3myc^ and Shp1^3HA^ as indicated were subjected to immunoprecipitation with anti-HA antibody and analyzed for co-precipitation of Glc7^3myc^. The asterisk marks the immunoglobulin heavy chain of the HA antibody. Quantification of the Glc7 signal in the IP lanes relative to the heavy chain signal revealed a more than eight-fold difference between the Shp1^3HA^ lane and the negative control. (e, f) Nuclear localization of Glc7^GFP^ in *shp1* mutants. WT, *shp1-7* and *shp1-b1* cells expressing Glc7^GFP^ as sole source of Glc7 were analyzed by confocal spinning disk microscopy. (e) Representative z-stack projections generated with ImageJ. Scale bars: 5 µm. (f) Quantification of the GFP signal in equal areas of nucleus *versus* cytosol in single z-slices of confocal images. (g) Normal binding of Glc7 to Sds22 in *shp1-7*. Lysates of WT and *shp1-7* cells expressing Sds22^3myc^ as indicated were subjected to immunoprecipitation with anti-myc antibody and analyzed for co-precipitation of untagged Glc7. The asterisk marks the immunoglobulin light chain of the myc antibody.

In order to investigate potential reasons for the reduced Glc7 activity in *shp1* mutants, we analyzed Glc7 protein levels and subcellular localization using epitope-tagged Glc7 variants. Because both over-expression and epitope-tagging of Glc7 can affect viability [32], [83], [84], we generated strains expressing carboxyl-terminally tagged Glc7 from its authentic chromosomal locus as the sole source of Glc7 activity. Based on their normal growth at 30°C and 37°C, we concluded that cells expressing Glc7^GFP^ and Glc7^3myc^ are not defective in critical aspects of Glc7 function in the DF5 strain background (Fig. 7a). In contrast, expression of Glc7^3HA^ causes a partial-loss-of-function phenotype reflected in temperature-sensitive growth. The functionality of the Glc7^GFP^ fusion protein was further confirmed by flow cytometry revealing a wild-type cell cycle distribution (Fig. 7b).

Using the functional, epitope-tagged Glc7^3myc^ protein we were able to show a physical interaction between Glc7 and Shp1 at endogenous expression levels by immunoprecipitation for the first time (Fig. 7c). The interaction was confirmed in a reciprocal experiment, where Glc7^3myc^ was co-immunoprecipitated with Shp1^3HA^ (Fig. 7d). This physical interaction between Shp1 and Glc7 could suggest that Shp1 directly controls the half-life or cellular localization of Glc7. Because we could not detect differences between wild-type and *shp1* cells in the protein levels of endogenous, untagged or epitope-tagged Glc7 (data not shown; see *e.g.* input lanes in Fig. 7g), we performed a thorough analysis of Glc7 subcellular localization by confocal spinning disk live-cell microscopy of cells expressing Glc7^GFP^ (Fig. 7e). Consistent with previous reports [84]–[89], the majority of Glc7^GFP^ was detected in the nucleus of wild-type cells, with additional diffuse cytosolic staining and a distinct localization at the septum of medium and large budded cells. *shp1-7* and *shp1-b1* cells showed a very similar distribution of Glc7^GFP^ with respect to nuclear, cytosolic, and septum localization, and no aberrant localization or aggregation of Glc7^GFP^ was observed (Fig. 7e; Figs. S2, S3). Quantification of the intensity of the nuclear *versus* cytosolic GFP signal revealed a slight decrease of nuclear Glc7 in the *shp1* mutants to approximately 80% of the wild-type signal (Fig. 7f). Interestingly, in the course of these experiments, it became evident that the nuclear localization of Glc7^GFP^ is influenced by the presence of untagged Glc7 in *shp1* mutants, but not wild-type cells. The additional expression of *GLC7* from a plasmid resulted in a notable reduction of the nuclear Glc7^GFP^ signal in *shp1-7 GLC7^GFP^* (Fig. S3). This effect appears to correlate with the expression level of untagged Glc7, as a high-copy plasmid encoding *GLC7* under control of the strong *ADH1* promoter reduced the nuclear Glc7^GFP^ signal even further (Fig. S3). It should be stressed, however, that lack of Shp1 clearly did not abolish nuclear localization of Glc7^GFP^ if this is the sole source of Glc7 (Fig. 7ef).

The nuclear localization of Glc7 was further assessed by analyzing the interaction of endogenous, untagged Glc7 with its nuclear targeting factor Sds22 [84], [86], [90]. Co-immunoprecipitation of Glc7 with Sds22 from lysates of wild-type and *shp1-7* strains demonstrated that the interaction between Glc7 and Sds22 was not reduced in *shp1-7* (Fig. 7g). Because the majority of Sds22 interacts with Glc7 in the nucleus [90], these data strongly suggest a normal nuclear localization of the Sds22-Glc7 complex in *shp1-7*. Taken together, our results make the possibility that the nuclear localization of Glc7 is grossly affected in *shp1* null mutants highly unlikely.

### The interaction between Glc7 and Glc8 is impaired in *shp1* mutants

**Figure 8:**
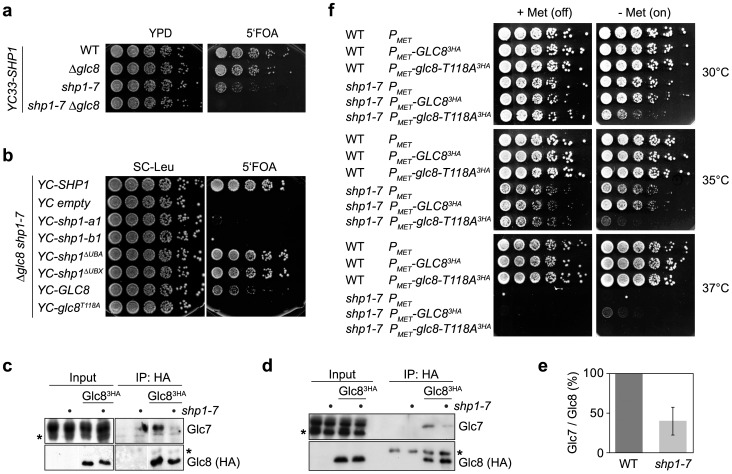
Impaired interaction between Glc7 and Glc8 in *shp1*. (a) Synthetic lethality of *shp1-7 Δglc8*. Growth of haploid progeny of one tetrad from the cross of *shp1-7* with *Δglc8* carrying *YC33-SHP1* was analyzed on control (YPD) and 5′FOA plates as described in the legend to Fig. 4b. (b) Synthetic lethality of *Δglc8* with *shp1* mutants defective in Cdc48 binding. *Δglc8 shp1-7* double mutant cells carrying *YC33-SHP1* and a *LEU2*-based centromeric plasmid for the expression of the indicated wild-type and mutant *SHP1* and *GLC8* alleles were analyzed as described in the legend to Fig. 4a. (c, d, e) Reduced physical interaction between Glc7 and Glc8 in *shp1-7*. Lysates of asynchronous (c) or α-factor-arrested (d) wild-type and *shp1-7* cells expressing Glc8^3HA^ were subjected to immunoprecipitation with anti-HA antibody and analyzed for co-precipitation of endogenous, untagged Glc7. The asterisks mark a cross-reactive band of the Glc7 antibody (Glc7 blots) and the immunoglobulin light chain of the HA antibody (Glc8 blots), respectively. In panel (c), irrelevant lanes were removed from the figure. However, all lanes shown were on the same Western blot and exposed and processed identically. (e) Quantification of three independent experiments as in panel (c), showing the ratio of the Glc7 and Glc8 signal intensities. (f) Overexpression of *GLC8* partially suppresses the temperature sensitivity of *shp1-7*. Wild-type (WT) and *shp1-7* cells expressing the indicated *GLC8^3HA^* alleles from an integrative plasmid under the control of the inducible *P_MET25_* promoter were analyzed for growth at the indicated temperatures in the presence (+Met (off)) and absence (−Met (on)) of methionine in the growth medium.

As neither protein levels nor cellular localization of Glc7 were severely affected in *shp1* mutants, we considered the possibility that Shp1 may influence the interaction of Glc7 with one or more of its numerous regulatory subunits. Because the physical interaction of Glc7 with its mitotic regulator Sds22 was unaltered in *shp1* (Fig. 7g), and no genetic interaction between *SHP1* and the putative Glc7 kinetochore recruitment factor *FIN1* [91] could be detected (data not shown), we next analyzed genetic interactions between *shp1* and *glc8* mutants (Fig. 8a). Glc8 is a positive regulator of Glc7, whose Glc7 activating function depends on phosphorylation of residue T118 by the cyclin-dependent kinase Pho85 [34], [92], [93]. Deletion of *GLC8* has been shown to cause a reduction of Glc7 phosphatase activity and cellular glycogen levels [94], but does not result in significant growth or cell cycle defects (Fig. 8a and data not shown). Some *glc7* alleles are synthetic lethal with *Δglc8* and *glc8* mutants that cannot be phosphorylated on residue T118 [93], indicating that activation by Glc8 becomes essential under conditions of reduced Glc7 activity. Interestingly, the *shp1-7 Δglc8* double mutant was found to be inviable as well (Fig. 8a), demonstrating that either Glc8 or Shp1 is required for viability of the DF5 strain background, presumably to ensure sufficient Glc7 activity. Consistent with this hypothesis, expression of *GLC8* restored growth of *shp1-7 Δglc8*, whereas the *glc8-T118A* allele was unable to rescue the synthetic lethality (Fig. 8b), proving that growth of *shp1-7* indeed requires the activation of Glc7 by Glc8. Similar to the genetic interaction between *shp1* and *glc7* (Fig. 4a), the ability of Shp1 to bind Cdc48 is required for viability of strains lacking *GLC8* (Fig. 8b), again demonstrating that the Cdc48^Shp1^ complex is necessary for normal regulation of Glc7 activity.

Glc8 has been demonstrated to physically interact with Glc7 *in vivo* and *in vitro* [92]. We therefore analyzed the effect of a *shp1* null mutation on the Glc7-Glc8 interaction by co-immunoprecipitation, using lysates from asynchronous and α-factor-arrested cultures of wild-type and *shp1-7 GLC8^3HA^* strains. The *shp1-7 GLC8^3HA^* strain was viable and did not exhibit any additional growth defect compared to *shp1-7* (data not shown), indicating that Glc8^3HA^ is functional. Intriguingly, the interaction of endogenous Glc7 with Glc8^3HA^ was significantly reduced in asynchronously growing as well as in G1-arrested *shp1-7* cultures (Fig. 8cd). Quantification of the Glc7 co-immunoprecipitation with Glc8 in asynchronous cultures of wild-type and *shp1-7* revealed that the Glc7-Glc8 interaction in *shp1-7* was reduced by approximately 50% (Fig. 8e). These data show that Shp1 is required for a normal physical interaction between Glc7 and its activator Glc8.

We next tested if the reduced interaction between Glc7 and Glc8 in *shp1-*7 is responsible for *shp1* phenotypes. To this end, we over-expressed *GLC8^3HA^* under the control of the *MET25* promoter (Fig. 8f). Importantly, the over-expression of *GLC8^3HA^* partially suppressed the growth defects of *shp1-7*, as improved growth at 35°C and weak, but detectable growth at the non-permissive temperature of 37°C was observed (Fig. 8f). In contrast, over-expression of *glc8-T118A^3HA^* was toxic in *shp1-7* cells (Fig. 8f), presumably because excess non-activating Glc8-T118 protein competed with endogenous Glc8 for Glc7 binding. In summary, our data show that the Cdc48^Shp1^ complex is important for the activation of Glc7 by Glc8, and that lack of Glc8-mediated activation contributes critically to the phenotype of *shp1* mutants.

## Discussion

This study addresses the relationship of Shp1, a major Cdc48 cofactor, and Glc7, the catalytic subunit of budding yeast PP1. We found that *shp1* mutants exhibit a variety of severe phenotypes, including a significant mitotic delay during progression from metaphase to anaphase. We were able to show that the mitotic phenotype of *shp1* mutants is caused by limiting nuclear Glc7 activity towards mitotic substrates, resulting in their hyper-phosphorylation due to unbalanced Ipl1 kinase activity. By engineering *shp1* alleles specifically defective in Cdc48 binding, we established that Shp1 regulates Glc7 in its capacity as a Cdc48 cofactor. Importantly, we could demonstrate that Shp1 and Glc7 interact physically, and that the Cdc48^Shp1^ complex is required for normal interaction of Glc7 with Glc8.

*shp1* mutants were originally found to exhibit reduced Glc7 activity towards glycogen phosphorylase, decreased glycogen accumulation, and defective sporulation [32]. Other *shp1* phenotypes attributed to reduced Glc7 activity include defective vacuolar degradation of fructose-1,6-bisphosphatase through the vacuole import and degradation (Vid) pathway [60], impaired V-ATPase activity [61], and impaired glucose repression [62]. Here, we provide several lines of evidence that *shp1* mutants also possesses a significant defect in mitotic Glc7 activity. First, the genetic interactions between *shp1* and *glc7*, *sds22*, *mad2*, and *ipl1* all point towards impaired nuclear function(s) of Glc7 in *shp1*. Second, over-expression of *GLC7* in *shp1* restored a normal cell cycle distribution and suppressed chromosome segregation defects. Third, the nuclear Glc7 substrates histone H3 and Dam1 are hyper-phosphorylated in *shp1* in an Ipl1-dependent manner. Together with the previously described cytosolic and vacuolar processes, the elucidation of its involvement in mitotic Glc7 functions underscores the importance of Shp1 as a positive regulator of many, if not most, Glc7 functions.

Dephosphorylation of Ipl1/Aurora B substrates at kinetochores is a well-established and evolutionarily conserved mitotic function of Glc7/PP1 [95]–[97]. Recently, however, Glc7 has also been implicated in silencing of the SAC [98]–[101], raising the possibility that impaired SAC silencing rather than chromosome attachment defects causes the mitotic delay of *shp1*. The observed stabilization of Pds1 in *shp1* (Fig. 3a) as well as the suppression of the mitotic delay by over-expression of *GLC7* (Fig. 5b) would in fact be consistent with both scenarios. However, the suppression of the chromosome segregation defects indicating defective chromosome bi-orientation by over-expression of *GLC7* (Fig. 5c) provides evidence that disturbed kinetochore-microtubule attachments are the primary cause for the mitotic delay in *shp1*. Furthermore, while *glc7* mutants defective in SAC silencing are rescued by deletion of *MAD2* [99], the pronounced negative genetic interaction observed for the *shp1-7 Δmad2* double mutant also strongly argues against a causative role of impaired SAC silencing for the mitotic phenotype of *shp1* (Fig. 3c). It rather shows that SAC inactivation/deletion in the continued presence of mitotic defects is highly detrimental to *shp1*. According to our data, the key mitotic defect of *shp1* mutants is the unbalanced Ipl1 activity at the kinetochore. This conclusion is not only supported by the positive genetic interaction between *shp1-7* and *ipl1-321*, but also underlined by the observed hyper-phosphorylation of the Ipl1 targets H3 and Dam1, which is suppressed in the *shp1-7 ipl1-321* double mutant.

At the kinetochore, the delicate balance between Ipl1 and Glc7 activities is believed to control cycles of association and dissociation of spindle microtubules that ultimately lead to proper bi-polar attachment and thus satisfaction of the SAC and mitotic progression [57], [58], [91]. The essential microtubule-binding protein Dam1 has been shown to be a critical target of Ipl1 [54], [55], [82] and Glc7 [56] during this process. Dam1 is the central component of the heterooligomeric Dam1/DASH complex located at the plus ends of spindle microtubules. There, the Dam1/DASH complex recruits the Ndc80 complex and thereby ensures dynamic coupling of microtubule plus ends with kinetochores [79], [80]. Of note, Ndc80 recruitment is abolished by Ipl1-mediated phosphorylation of Dam1 or by phospho-mimicking mutations in Ipl1 target sites of Dam1 [79], [80]. Importantly, our results show for the first time that Dam1 is hyper-phosphorylated in *shp1*, and that this altered modification significantly contributes to the severe phenotype of *shp1* mutants. Using the *dam1SA* and *damSD* alleles, we set out to mimic the effects of *ipl1* and *glc7* loss-of-function mutations, respectively, on this specific target. Intriguingly, altering the relative abundance of Dam1 phospho-sites in *shp1* almost perfectly phenocopied the genetic interactions of *shp1* with *ipl1* and *glc7*. Over-expression of *dam1SA* allowed robust growth up to 35°C similar to the *shp1-7 ipl1-321* double mutant, whereas over-expression of *dam1SD* was toxic, albeit this effect was less severe than that observed for the *shp1-7 glc7-129* double mutant. These results clearly show that Dam1 hyper-phosphorylation is a major cause of *shp1* phenotypes related to mitotic functions of Glc7 and Ipl1.

Our analysis of the mitotic phenotype of viable, logarithmically growing *shp1* mutant cells in the DF5 strain background is largely consistent with the results of a recent study using the temperature-sensitive *cdc48-3* allele and a *P_GAL_-3HA-SHP1* allele for the conditional depletion of Shp1 in the W303 strain background [31]. The authors of that study concluded that Cdc48 and Shp1 are important for the kinase to phosphatase balance at the kinetochore and proposed that Cdc48^Shp1^ regulates the nuclear localization of Glc7. Our study goes beyond their analysis and differs in certain central aspects. We were able to demonstrate strong positive and negative genetic interactions, respectively, of *shp1* null and Cdc48 binding-deficient alleles with *ipl1-321* and *glc7-129*. Importantly, we proved that the cell cycle and chromosome segregation defects of *shp1* null and Cdc48 binding-deficient mutants are efficiently suppressed by increased Glc7 levels. Finally, we established an increased Dam1 phosphorylation in *shp1* mutants, which can be suppressed by a reduction of Ipl1 activity.

One likely explanation for the differences between the two studies relates to the strains used by Cheng and Chen. In particular, the use of the *cdc48-3* strain poses problems due to its pleiotropic phenotypes. Besides defects in the kinetochore-microtubule attachment reported by Cheng and Chen, *cdc48-3* has been shown to be impaired in G1 progression [64], [66], spindle disassembly at the end of mitosis [65], transcription factor remodeling [102], UV-induced turnover of RNAPolII [24], ERAD [103], [104], and autophagy [30]. As long as specific targets of Cdc48 at the kinetochore remain unknown, it is therefore almost impossible to differentiate between direct and secondary effects of the *cdc48-3* allele on cell cycle progression. Furthermore, Cheng and Chen state that the observed mitotic phenotypes of *cdc48-3* were generally more severe than those of Shp1-depleted cells. This finding is likely to reflect the involvement of alternative Cdc48 cofactors, in particular Ufd1-Npl4, in Shp1-independent functions of Cdc48 during the cell cycle. Taken together, the uncertainties in the interpretation of *cdc48-3* phenotypes underscore the importance of designing specific Cdc48 binding-deficient *shp1* alleles. The *shp1* alleles presented in this study enabled us to study genetic interactions and the effect of *GLC7* over-expression in the absence of unrelated pleiotropic defects and thus allowed us to formally conclude for the first time that the regulation of Glc7 activity indeed requires the Cdc48^Shp1^ complex.

The major discrepancy between this study and the study by Cheng and Chen relates to the cellular localization of Glc7 in the absence of Shp1. While these authors found that depletion of Shp1 leads to the loss of Glc7 accumulation in the nucleus, our microscopy data of strains expressing a fully functional Glc7^GFP^ fusion protein as the sole source of Glc7 indicated only a moderate reduction of nuclear Glc7 in *shp1* (Fig. 7ef). These data are supported by a normal co-immunoprecipitation of Glc7 with its nuclear targeting subunit Sds22 in *shp1* (Fig. 7g), and they are in agreement with data from biochemical fractionation experiments [32]. There are two potential explanations for the discrepancy of our data with those by Cheng and Chen. First, we found that the nuclear localization of Glc7^GFP^ in *shp1* is reduced in the presence of additional, untagged Glc7 (Fig. S3) for unknown reasons. Cheng and Chen used a strain expressing ^GFP^Glc7 in addition to endogenous Glc7, raising the possibility that these conditions prevented a nuclear localization of the tagged Glc7 variant. Second, Cheng and Chen performed microscopy 12 hours after promoter shut-off under conditions of ongoing cell death, whereas our analysis was performed with logarithmically growing *shp1* cells. Altogether, considering the available experimental evidence, a gross reduction of nuclear Glc7 levels in *shp1* null mutants appears unlikely. In line with this conclusion, cytoplasmic Glc7 functions in glycogen metabolism and in the Vid pathway are affected in *shp1* mutants as well [32], [60], also arguing against impaired nuclear localization of Glc7 as the critical defect in *shp1*.

Besides the genetic interactions between *glc7* and *shp1* mutants, the present study showed for the first time that Shp1 and Glc7 also interact physically (Fig. 7cd). We currently do not know if this interaction is direct or indirect, for instance bridged by regulatory subunits of Glc7. While Shp1 lacks a classical RVxF motif (data not shown), which mediates the binding of many PP1 regulatory subunits [34], [105], [106], a number of Glc7 subunits interact through other motifs (reviewed in [34], [106]). Alternatively, Cdc48^Shp1^ could interact with ubiquitylated Glc7 or an ubiquitylated Glc7 interactor. Consistent with this possibility, we found that Glc7 is ubiquitylated *in vivo* (data not shown), in agreement with proteomics studies [107]–[109]. Clearly, the molecular basis for Shp1 binding to Glc7 remains to be elucidated in future studies.

The identification of a physical interaction between Shp1 and Glc7 raises the intriguing possibility that Cdc48^Shp1^ controls Glc7 cellular functions by modulating binding of regulatory subunits. While we failed to detect Shp1-dependent differences in the interactions of Glc7 with Sds22 (Fig. 7g) and Reg1 (data not shown; see [60]), we found a strikingly reduced binding between Glc7 and Glc8 in *shp1* (Fig. 8cde). Because Glc8 is considered a substrate-independent, major activator of Glc7, the reduced interaction could at least partially explain the broad spectrum of Glc7 functions affected in *shp1* mutants. This interpretation is strengthened by the finding that *GLC8* over-expression partially suppressed the temperature-sensitivity of *shp1* (Fig. 8f). However, the reduced binding of Glc8 to Glc7 cannot be the sole cause of the pleiotropic Glc7-related phenotypes of *shp1*. The much less severe phenotypes of *Δglc8* clearly show that *GLC8* is not strictly required for viability in an otherwise unperturbed cell, suggesting that more complex mechanisms for the positive regulation of Glc7 activity must exist. Furthermore, the synthetic lethality of *shp1* and *Δglc8* (Fig. 8a) cannot be explained on basis of the reduced interaction between Glc7 and Glc8 observed in the *shp1-7* single mutant. We therefore favor the hypothesis that the Cdc48^Shp1^ complex controls the balance of Glc7 interactions with additional regulatory subunits, perhaps by mediating the dissociation of certain regulatory subunits from Glc7 by virtue of the segregase mechanism underlying other cellular functions of Cdc48 [3]–[5]. Interestingly, regulatory subunits appear to exist in excess over Glc7 *in vivo* [110], suggesting that they compete for binding to Glc7 [34]. In support of a competitive model for Glc7 binding, over-expression of several regulatory subunits has been shown to re-direct cellular Glc7 activity [56], [111]. We speculate that in such a scenario of competitive subunit interactions, loss of Cdc48^Shp1^ segregase activity would stabilize the interaction of certain regulatory subunits with Glc7, resulting in reduced binding of Glc8 and additional, yet unknown, subunits required for mitotic progression. The future identification of additional Cdc48^Shp1^ targets involved in Glc7 regulation will be critical for the experimental evaluation of this hypothesis.

## Materials and Methods

### Plasmids

Plasmids used in this study are listed in Table 1. Unless specified otherwise below, genomic fragments of wild-type and mutant alleles were PCR-amplified from genomic yeast DNA or plasmids and cloned into yeast shuttle vectors [112] using standard techniques; details are available upon request from the authors. For the construction of *YEplac195-P_ADH_-*GLC7, the *GLC7* coding region was PCR-amplified from cDNA and cloned *via* BamHI/PstI into YEplac195 [112] modified to carry the *ADH1* promoter and terminator in its EcoRI/BamHI and PstI/SphI sites, respectively (pAB1376). For the construction of *YIplac128-P_MET25_-GLC7*, the *P_MET25_* promoter was subcloned from pUG36 [113] into the SacI and XbaI sites of YIplac128 [112] modified to carry the coding sequence for a carboxyl-terminal 3HA epitope tag and the *ADH1* terminator (PstI/NlaIII fragment of pYM1 [114] subcloned into YIplac128 *via* PstI/SphI) (pAB1165). The *GLC7* coding sequence including the stop codon was cloned into pAB1165 *via* SpeI/PstI (pAB1280). The coding sequences (excluding the stop codon) of *GLC8* or *glc8-T118A* were cloned into pAB1165 *via* XbaI/PstI. *YIplac211-GFP-LacI* was constructed by subcloning the *P_HIS3_-GFP-LacI-NLS* fragment from pAFS135 [115] into YIplac211 [112] *via* KpnI/XbaI (pAB2040). *shp1* mutant alleles including *shp1-7* (first codon mutated from *ATG* to *ACC*) and alleles carrying combinations of mutations in Cdc48 binding sites as depicted in Fig. 2a (F396/P397/I398→G; R360→A; F306/Q309/Q311/R312/L313→AAAAA; F201/R204/F206/R207/L208→AAAAA; details available from the authors upon request) were generated from pAB1847 by site-specific mutagenesis using the QuikChange II XL kit (Stratagene) and verified by sequencing.

**Table 1:** Plasmids used in this study.

Plasmid	Description	Source
pAB827	*YCplac33-SHP1*	[20]
pAB1808	*YCplac22-SHP1*	this work
pAB855	*YCplac111-SHP1*	[123]
pAB1845	*YCplac111-shp1-a1*	this work
pAB1795	*YCplac111-shp1-b1*	this work
pAB856	*YCplac111-shp1^ΔUBA^*	[123]
pAB857	*YCplac111-shp1^ΔUBX^*	[123]
pAB1785	*YCplac111-GLC7*	this work
pAB1756	*YCplac111-GLC8*	this work
pAB1757	*YCplac111-glc8^T118A^*	this work
pAB1376	*YEplac195-P_ADH_-GLC7*	this work
pAB1740	*YEplac195-SDS22*	[41]
pAB1887	*YEplac112-DAM1*	this work
pAB1888	*YEplac112-dam1SA* (S20A, S292A)	this work
pAB1889	*YEplac112-dam1SD* (S20D, S292D)	this work
pAB1165	*YIplac128-P_MET25_*	this work
pAB1280	*YIplac128-P_MET25_-GLC7*	this work
pAB1745	*YIplac128-P_MET25_-GLC8^3HA^*	this work
pAB1746	*YIplac128-P_MET25_-glc8T-118A^3HA^*	this work
pAB1847	*YIplac211-SHP1*	this work
pAB1784	*YIplac211-shp1-7*	this work
pAB1796	*YIplac211-shp1-b1*	this work
pAB1805	*YIplac211-shp1-a1*	this work
pAB1945	*YIplac211-shp1-a3*	this work
pAB1946	*YIplac211-shp1-a4*	this work
pAB1947	*YIplac211-shp1-a5*	this work
pAB1818	*YIplac211-shp1^ΔUBA^*	this work
pAB1819	*YIplac211-shp1^ΔUBX^*	this work
pAFS59	*YIplac128-256xlacO*	[115]
pAB2040	*YIplac211-^GFP^LacI*	this work

### Yeast strains and media

All strains used in this study are derivatives of DF5 [116] and listed in Table 2. Chromosomal deletions and fusions with epitope tags or fluorescent proteins were generated using standard methods [114], [117], [118]. For the characterization of Cdc48 binding-deficient *shp1* alleles (Fig. 2a–c), the respective *YIplac211-shp1* plasmids were linearized in *URA3* with StuI and transformed into *Δshp1::kanMX6*. Transformants were selected on SC-Ura media for correct integration into the *ura3-52* locus, and expression of Shp1 was confirmed by Western blot. For the construction of the *shp1*-7, *shp1-a1* and *shp1-b1* mutant strains by a pop-in pop-out strategy, the respective *YIplac211-shp1* plasmids were linearized within the *SHP1* open reading frame by restriction digest with BamHI and transformed into DF5a. Transformants were selected for Ura prototrophy, and correct integration was verified by colony PCR. Positive clones were streaked out twice on 5′FOA, and single colonies were picked. These clones were then analyzed by colony PCR for the presence of the full-length *shp1* allele to exclude aberrant pop-out events, by Western blot for Shp1 levels, and for temperature sensitivity. Similar approaches were used to transfer the conditional alleles *glc7-129*, *ipl1-321*, and *sds22-6* into the DF5 strain background. All mutant alleles were finally sequenced, either by cloning the open reading frame together with 1 kb of upstream and downstream flanking sequences, or by directly sequencing a PCR product of the mutated region. Double mutants were constructed by crossing the respective conditional allele with the *shp1-7* mutant carrying *YCplac33-SHP1*.

**Table 2:** Yeast strains used in this study.

Strain	Genotype	Source
DF5a	MATa *ura3-52, leu2-3,-112 lys2-801, trp1-1, his3Δ200*	[116]
YAB589	DF5a *Δshp1::kanMX6*	[5]
YAB1729	DF5a *shp1-7*	this work
YAB1568	DF5a *shp1-b1*	this work
YAB1564	DF5a *shp1-a1*	this work
YAB1288	DF5a *Δshp1::kanMX6 YIplac211-shp1-a1::URA3*	this work
YAB1712	DF5a *Δshp1::kanMX6 YIplac211-shp1-a3::URA3*	this work
YAB1713	DF5a *Δshp1::kanMX6 YIplac211-shp1-a4::URA3*	this work
YAB1714	DF5a *Δshp1::kanMX6 YIplac211-shp1-a5::URA3*	this work
YAB1276	DF5a *Δshp1::kanMX6 YIplac211-shp1^ΔUBA^::URA3*	this work
YAB1275	DF5a *Δshp1::kanMX6 YIplac211-shp1^ΔUBX^::URA3*	this work
YAB1422	DF5a *CLN2^3HA^::klTRP1*	this work
YAB1423	DF5a *Δshp1::kanMX6 CLN2^3HA^::klTRP1*	this work
YAB1378	DF5alpha *SPC42^Mars^:: nat-NT2*	this work
YAB1383	DF5a *Δshp1::kanMX6 SPC42^Mars^::nat-NT2*	this work
YAB1642	DF5a *PDS1^18myc^::klTRP1*	this work
YAB1643	DF5a *shp1-7 PDS1^18myc^::klTRP1*	this work
YAB1585	DF5a *Δmad2::HIS3MX6*	this work
YAB1582	DF5a *shp1-7 Δmad2::HIS3MX6*	this work
YAB1469	DF5a *GLC7^3myc^::HIS3MX6 SHP1^3HA^::klTRP1*	this work
YAB1464	DF5a *GLC7^3myc^::HIS3MX6*	this work
YAB1466	DF5a *Δshp1::kanMX6 GLC7^3myc^::HIS3MX6*	this work
YAB1470	DF5a *SHP1^3HA^:: klTRP1*	this work
YAB1447	DF5a *GLC7^3HA^::klTRP1*	this work
YAB1587	DF5α *glc7-129*	this work
YAB1607	DF5α *shp1-7 glc7-129 YC33-SHP1*	this work
YAB1611	DF5α *ipl1-321*	this work
YAB1736	DF5 *shp1-7 ipl1-321*	this work
YAB1451	DF5a *DAM1^9myc^::HIS3MX6*	this work
YAB1656	DF5α *ipl1-321 DAM1^9myc^::klTRP1*	this work
YAB1655	DF5α *glc7-129 DAM1^9myc^::klTRP1*	this work
YAB1657	DF5 *shp1-7 ipl1-321 DAM1^9myc^::klTRP1*	this work
YAB1496	DF5a *YIplac128-P_MET25_::LEU2*	this work
YAB1738	DF5a *shp1-7 YIplac128-P_MET25_::LEU2*	this work
YAB1473	DF5a *YIplac128-P_MET25_-GLC7::LEU2*	this work
YAB1731	DF5a *shp1-7 YIplac128-P_MET25_-GLC7::LEU2*	this work
YAB1660	DF5a *shp1-a1 YIplac128-P_MET25_::LEU2*	this work
YAB1661	DF5a *shp1-a1 YIplac128-P_MET25_-GLC7::LEU2*	this work
YAB1445	DF5a *GLC7^GFP^::klTRP1*	this work
YAB1538	DF5a *shp1-7 GLC7^GFP^::klTRP1*	this work
YAB1603	DF5a *shp1-b1 GLC7^GFP^::klTRP1*	this work
YAB1494	DF5α *Δglc8::hph-NT1*	this work
YAB1647	DF5 *shp1-7 Δglc8::hph-NT1 YCplac33-SHP1*	this work
YAB1499	DF5a *SDS22^3myc^::klTRP1*	this work
YAB1555	DF5a *shp1-7 SDS22^3myc^::klTRP1*	this work
YAB1553	DF5a *GLC8^3HA^*::*klTRP1*	this work
YAB1554	DF5a *shp1-7 GLC8^3HA^*::*klTRP1*	this work
YAB1430	DF5a *Δbar1::hph-NT1*	this work
YAB1596	DF5a *shp1-7 Δbar1::hph-NT1*	this work
YAB1598	DF5a *GLC8^3HA^*::*klTRP1 Δbar1::hph-NT1*	this work
YAB1599	DF5a *shp1-7 GLC8^3HA^*::*klTRP1 Δbar1::hph-NT1*	this work
YAB1664	DF5a *YIplac128-P_MET25_-GLC8^3HA^::LEU2*	this work
YAB1665	DF5a *YIplac128-P_MET25_-glc8-T118A^3HA^::LEU2*	this work
YAB1667	DF5a *shp1-7 YIplac128-P_MET25_-GLC8^3HA^::LEU2*	this work
YAB1668	DF5a *shp1-7 YIplac128-P_MET25_-glc8-T118A^3HA^::LEU2*	this work
YAB1703	DF5α*sds22-6 YCplac33-SHP1*	this work
YAB1705	DF5α*shp1-7 sds22-6 YCplac33-SHP1*	this work
YAB1458	DF5a *POM34^Mars^::nat-NT2 GLC7^GFP^::kl-TRP1*	this work
YAB1601	DF5a *shp1-7 POM34^Mars^::nat-NT2 GLC7^GFP^::kl-TRP1*	this work
YAB1770	DF5a *shp1-b1 POM34^Mars^::hph-NT1 GLC7^GFP^::kl-TRP1*	this work
YAB1771	DF5a *SPC42^Mars^::nat-NT2 YIplac128-256xlacO::LEU2 YIplac211-^GFP^LacI::HIS3::ura3 YIplac128-P_MET25_-GLC7::GLC7*	this work
YAB1772	DF5a *shp1-7 SPC42^Mars^::nat-NT2 YIplac128-256xlacO::LEU2 YIplac211-^GFP^LacI::HIS3::ura3 YIplac128-P_MET25_-GLC7::GLC7*	this work
YAB1773	DF5a *shp1-a1 SPC42^Mars^::nat-NT2 YIplac128-256xlacO::LEU2 YIplac211-^GFP^LacI::HIS3::ura3 YIplac128-P_MET25_-GLC7::GLC7*	this work

Yeast was cultured in standard YPD and SC media [119]. For the induction of the *P_MET25_* promoter, cells were first grown in SC media supplemented with 2 mM methionine, washed twice with H_2_O, and then transferred to SC medium lacking methionine.

### α-factor arrest/release

Overnight cultures of wild-type and mutant strains were diluted to an OD_600 nm_ of 0.1 (0.15 for *shp1* mutants) in 50 ml YPD. The cultures were then grown at 25°C for approximately four hours until reaching an OD_600 nm_ of 0.3–0.35. 10 µM α-factor (central core facility, Max Planck Institute of Biochemistry, Martinsried, Germany) in DMSO were added, and the cells were allowed to arrest for three hours at 25°C. Directly before addition of α-factor, a control sample from the asynchronous culture was collected, and the pellet was frozen in liquid nitrogen. The efficiency of the arrest was determined by FACS analysis and/or Western blot for Clb2 levels after three hours of arrest. The cultures were then washed two times with equal volumes of YPD and resuspended to a final OD_600 nm_ of approximately 0.5 in YPD. The released cultures were incubated at 25°C, and at various time-points an amount of cells corresponding to 1 ml of OD_600 nm_ = 0.6 was removed from the culture, quickly pelleted and frozen in liquid nitrogen. Subsequently, the cell pellets were lysed by TCA precipitation and resuspended in 50 µl HU/DTT buffer (8 M urea, 5% SDS, 0.2 M Tris pH 6.8, bromophenol blue, 0.1 M DTT). Fluctuations in the levels of Clb2 and other cell cycle marker proteins were analyzed by Western blot.

### Antibodies

Affinity-purified rabbit polyclonal antibodies against Shp1 [20], Cdc48 [120] and Glc7 [121] were described previously. The following commercially available antibodies were used: myc (9E10; M5546, Sigma), HA (F7, Santa Cruz), GFP (JL-8, Clontech), Clb2 (y-180, Santa Cruz), mammalian histone H3 ChIP grade (ab1791, Abcam), phospho-H3 (06-570, Upstate).

### FACS analysis

Analysis of DNA content by FACS was performed exactly as described [120] using a BD FACS Calibur and CellQuest Pro software or a BD FACS Canto and FACS Diva software.

### Microscopy

Yeast strains were grown in appropriate, sterile-filtered SC media to avoid high background fluorescence. Cells were immobilized by incubation on cover slips coated with 1 mg/ml concanavalin A (Type 5, Sigma Aldrich) for at least 30 min. Cells from logarithmically growing cultures were directly spotted on the cover slip, shortly incubated and sealed with Vaseline. Spinning disk confocal microscopy of Glc7^GFP^ localization (Fig. 7e) was performed using a microscope setup described previously [122]. In brief, cells expressing Glc7^GFP^ were analyzed using a laser-based spinning disk confocal microscope (Andor Technology). Filtered images (Semrock emission filters in a Sutter filter wheel) were captured with a D-977 iXon EMCCD+ camera (Andor Technology) after twofold magnification (Andor Technology) by using a 100×TIRFM/1.45 objective (Olympus). Z-Stacks were recorded with a spacing of 0.2 µm over the entire cell (10–25 planes). Images were processed with ImageJ software (http://rsbweb.nih.gov/ij/) and the MBF ImageJ for Microscopy collection of plug-ins (http://www.macbiophotonics.ca/imagej/). For quantification of the Glc7^GFP^ signal, single Z-slices of confocal images that had been recorded under identical conditions were used. The average GFP fluorescence intensity was measured in an area of equal size in the nucleus and cytoplasm using ImageJ software, and the ratio was calculated. Fluorescence microscopy of Glc7^GFP^ localization upon additional expression of untagged *GLC7* (Fig. S3) was carried out using a Zeiss Axiovert 200 M microscope equipped with an Axio Apochrom (Zeiss) 100×/1.4 oil objective and the filter set #10 (FITC). Images were captured using an AxioCam MRm TV2/3″ 0.63× (Zeiss) camera and AxioVision LE software.

For the analysis of sister chromatid separation (Fig. 5cd), cultures were grown to log-phase in SC medium+/−2 mM methionine, harvested, and resuspended in sterile filtered medium. 1.4% low-melting agarose was added in equal volume to mount the samples on cover slips. Microscopy was carried out on a Nikon TiE inverted live cell system with a motorized Prior Z-stage and Perfect Focus System using a 100× 1.45 NA objective (Nikon). Eleven Z-Stacks (spacing 0.3 µm) were recorded with a Photometrics HQ2 camera and analyzed using Nikon NIS Elements software. For differential interference contrast (DIC) microscopy, a single snap-shot was taken. All images were recorded using identical exposure times. Medium- to large-budded cells in each class were counted using NIS Elements software by assessing the position of the ^GFP^LacI-marked chromosome III relative to the SPBs (Spc42^Mars^).

### Immunoprecipitation

Yeast cultures were grown in YPD to an OD_600_ of 0.7, harvested and washed once with cold ddH_2_O/1 mM PMSF. Cells were then lysed in IP buffer (50 mM Tris/HCl pH 7.5, 100 mM KCl, 5 mM MgCl2, 0.1% NP-40, 10% glycerol, 10 mM NaF, 2 mM PMSF, complete protease inhibitor cocktail (Roche)) by addition of zirconia beads (Biospec) and vortexing. After lysis, the NP-40 concentration was raised to 1%, and the extracts were centrifuged at 2,600 g for 5 min, followed by centrifugation at 20,000 g for 25 min. An input sample (10 µl) was taken prior to antibody addition and denatured by addition of an equal amount of HU/DTT buffer and incubation at 65°C for 10 min. The supernatants were incubated with 20 µl pre-coupled HA antibody, 4.5 µl myc antibody, or 2 µl Shp1 antibody and rotated at 4°C overnight. Immunocomplexes were then either bound to 20 µl protein A sepharose beads (GE Healthcare) for three hours (4°C), or directly washed (pre-coupled HA antibody) four times (600 µl IP buffer/1% NP-40 for 10 min, 800 µl IP buffer/1% NP-40 8 min, 800 µl IP-buffer 5 min, 1 ml IP buffer). Bound proteins were eluted by incubation with 25 µl HU/DTT buffer for 10 min at 65°C and analyzed by Western blot.

## Supporting Information

Figure S1 Genetic interactions of *shp1* with *glc7* and *ipl1*. (a) Synthetic lethality of *shp1-7 glc7-129*. Growth of haploid progeny of one tetrad from the crossing of *shp1-7* with *glc7-129* carrying *YC33-SHP1* was analyzed on control (YPD) and 5′FOA plates as described in the legend to Fig. 4b. (b) Positive genetic interaction between *shp1-7* and *ipl1-321*. Growth of haploid progeny of one tetrad from the crossing of *shp1-7* with *ipl1-321* was analyzed at the indicated temperatures.
(TIF)

Figure S2 Nuclear localization of Glc7 in *shp1* mutants. Asynchronous logarithmic cultures of wild-type or the indicated *shp1* mutants expressing Glc7^GFP^ as the only source of Glc7 and the nuclear envelope marker Pom34^Mars^ were grown at RT and analyzed by live-cell fluorescence microscopy. Scale bar 5 µm. Fluorescent images are z-stack projections, DIC a single image of the focus plane.
(TIF)

Figure S3 *GLC7* expression levels influence the nuclear localization of the Glc7^GFP^ fusion protein in *shp1-7*. (a) Wild-type (WT) or *shp1-7* strains expressing Glc7^GFP^ as the only source of Glc7 were transformed with either empty YC plasmids or plasmids encoding the *GLC7* gene under control of its own promoter (*YC-GLC7*) or the *ADH* promoter (*YEpADH-GLC7*). Asynchronous logarithmic cultures of the indicated strains were analyzed by live-cell fluorescence microscopy. GFP (Glc7) fluorescence, DIC images, and the overlay are depicted. (b) Lysates of the cultures used in (a) were analyzed by Western blot against Glc7 and Cdc48 (loading control). For comparison, WT and *shp1-7* expressing endogenous untagged Glc7 are also shown. The asterisk marks a cross-reactive band of the Glc7 antibody.
(TIF)
