# Correction: The Budding Yeast Cdc48^Shp1^ Complex Promotes Cell Cycle Progression by Positive Regulation of Protein Phosphatase 1 (Glc7)

**DOI:** 10.1371/annotation/dfc860c8-7989-48f4-98e7-95b927d932c5

**Published:** 2013-10-01

**Authors:** Stefanie Böhm, Alexander Buchberger

There is an error in the Funding section. 

The correct funding information is as follows: This work was supported by grants BU951/3-1, BU951/3-2 and BU951/4-1 by the German Research Foundation (DFG) (http://www.dfg.de/en/index.jsp) to A.B. and the University of Wuerzburg in the funding programme Open Access Publishing. The funders had no role in study design, data collection and analysis, decision to publish, or preparation of the manuscript. 

