# Supplementary figures and images for: The Budding Yeast Cdc48Shp1 Complex Promotes Cell Cycle Progression by Positive Regulation of Protein Phosphatase 1 (Glc7)
Source: PLoS One. 2013 Feb 13;8(2):e56486. doi: 10.1371/journal.pone.0056486 (PMC3572051; doi:10.1371/journal.pone.0056486)

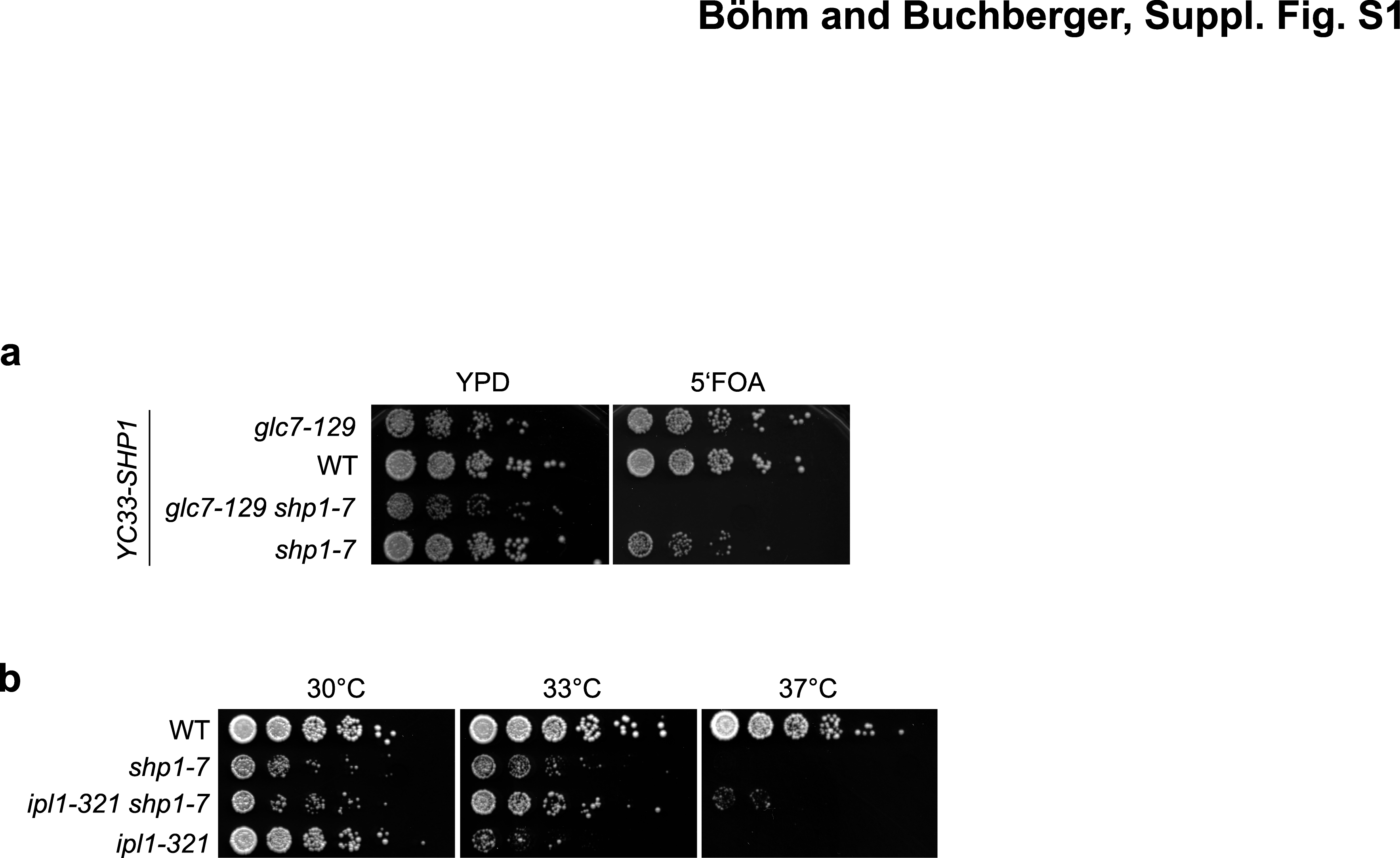

Supplement: Figure S1 — Genetic interactions of shp1 with glc7 and ipl1. (a) Synthetic lethality of shp1-7 glc7-129. Growth of haploid progeny of one tetrad from the crossing of shp1-7 with glc7-129 carrying YC33-SHP1 was analyzed on control (YPD) and 5′FOA plates as described in the legend to Fig. 4b. (b) Positive genetic interaction between shp1-7 and ipl1-321. Growth of haploid progeny of one tetrad from the crossing of shp1-7 with ipl1-321 was analyzed at the indicated temperatures. (TIF) [file pone.0056486.s001.tif]

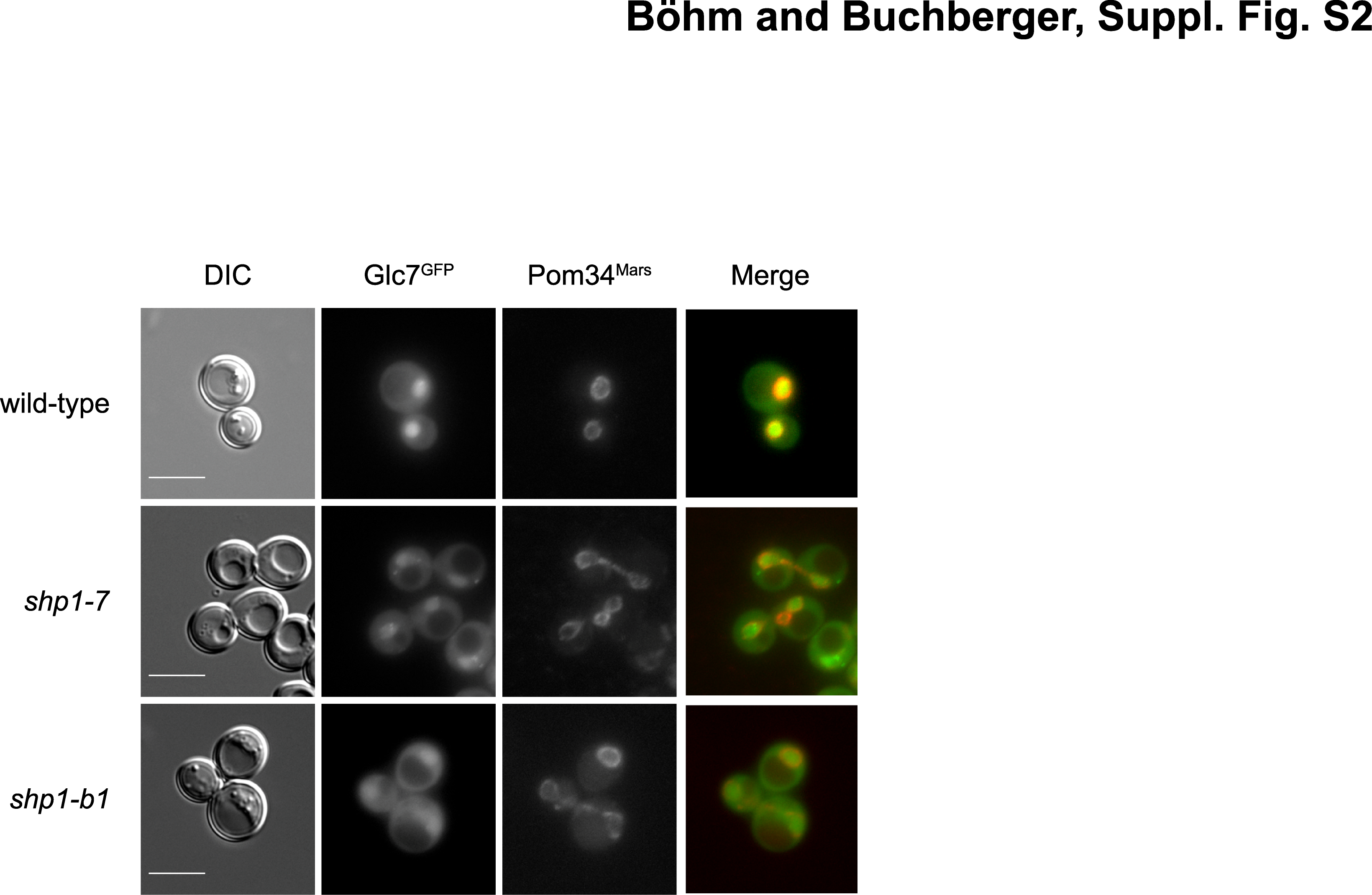

Supplement: Figure S2 — Nuclear localization of Glc7 in shp1 mutants. Asynchronous logarithmic cultures of wild-type or the indicated shp1 mutants expressing Glc7GFP as the only source of Glc7 and the nuclear envelope marker Pom34Mars were grown at RT and analyzed by live-cell fluorescence microscopy. Scale bar 5 µm. Fluorescent images are z-stack projections, DIC a single image of the focus plane. (TIF) [file pone.0056486.s002.tif]

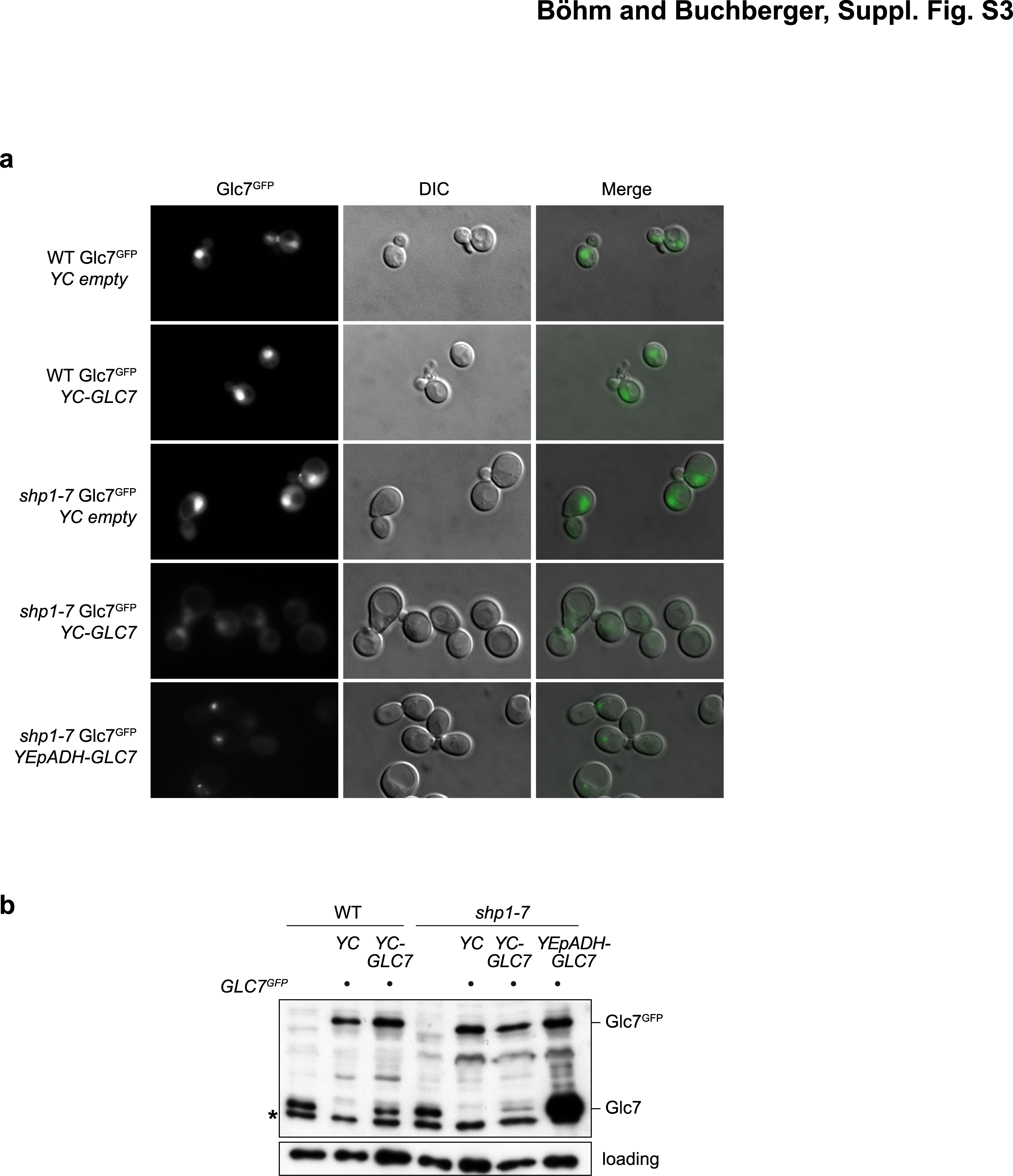

Supplement: Figure S3 — GLC7 expression levels influence the nuclear localization of the Glc7GFP fusion protein in shp1-7. (a) Wild-type (WT) or shp1-7 strains expressing Glc7GFP as the only source of Glc7 were transformed with either empty YC plasmids or plasmids encoding the GLC7 gene under control of its own promoter (YC-GLC7) or the ADH promoter (YEpADH-GLC7). Asynchronous logarithmic cultures of the indicated strains were analyzed by live-cell fluorescence microscopy. GFP (Glc7) fluorescence, DIC images, and the overlay are depicted. (b) Lysates of the cultures used in (a) were analyzed by Western blot against Glc7 and Cdc48 (loading control). For comparison, WT and shp1-7 expressing endogenous untagged Glc7 are also shown. The asterisk marks a cross-reactive band of the Glc7 antibody. (TIF) [file pone.0056486.s003.tif]
